# Evolution of surrogate light chain in tetrapods and the relationship between lengths of CDR H3 and VpreB tails

**DOI:** 10.3389/fimmu.2022.1001134

**Published:** 2022-10-13

**Authors:** Jeannine A. Ott, Jeremy K. Haakenson, Abigail R. Kelly, Claire Christian, Michael F. Criscitiello, Vaughn V. Smider

**Affiliations:** ^1^ Comparative Immunogenetics Lab, Department of Veterinary Pathobiology, School of Veterinary Medicine and Biomedical Sciences, Texas A&M University, College Station, TX, United States; ^2^ Applied Biomedical Science Institute, San Diego, CA, United States; ^3^ Department of Molecular Medicine, The Scripps Research Institute, La Jolla, CA, United States

**Keywords:** VpreB, Lambda5, pre-B cell receptor, surrogate light chain, pre-T cell receptor alpha, evolution

## Abstract

In the mammalian immune system, the surrogate light chain (SLC) shapes the antibody repertoire during B cell development by serving as a checkpoint for production of functional heavy chains (HC). Structural studies indicate that tail regions of VpreB contact and cover the third complementarity-determining region of the HC (CDR H3). However, some species, particularly bovines, have CDR H3 regions that may not be compatible with this HC-SLC interaction model. With immense structural and genetic diversity in antibody repertoires across species, we evaluated the genetic origins and sequence features of surrogate light chain components. We examined tetrapod genomes for evidence of conserved gene synteny to determine the evolutionary origin of VpreB1, VpreB2, and IGLL1, as well as VpreB3 and pre-T cell receptor alpha (PTCRA) genes. We found the genes for the SLC components (VpreB1, VpreB2, and IGLL1) only in eutherian mammals. However, genes for PTCRA occurred in all amniote groups and genes for VpreB3 occurred in all tetrapod groups, and these genes were highly conserved. Additionally, we found evidence of a new VpreB gene in non-mammalian tetrapods that is similar to the VpreB2 gene of eutherian mammals, suggesting VpreB2 may have appeared earlier in tetrapod evolution and may be a precursor to traditional VpreB2 genes in higher vertebrates. Among eutherian mammals, sequence conservation between VpreB1 and VpreB2 was low for all groups except rabbits and rodents, where VpreB2 was nearly identical to VpreB1 and did not share conserved synteny with VpreB2 of other species. VpreB2 of rabbits and rodents likely represents a duplicated variant of VpreB1 and is distinct from the VpreB2 of other mammals. Thus, rabbits and rodents have two variants of VpreB1 (VpreB1-1 and VpreB1-2) but no VpreB2. Sequence analysis of VpreB tail regions indicated differences in sequence content, charge, and length; where repertoire data was available, we observed a significant relationship between VpreB2 tail length and maximum DH length. We posit that SLC components co-evolved with immunoglobulin HC to accommodate the repertoire – particularly CDR H3 length and structure, and perhaps highly unusual HC (like ultralong HC of cattle) may bypass this developmental checkpoint altogether.

## Introduction

Before transcription of functional antibody genes can occur, immunoglobulin (Ig) gene segments first must be rearranged productively into functional genes. Recombination activating genes (RAG) mediate the recombination of V, D, and J gene segments of the Ig heavy chain locus and V and J gene segments of the Ig light chain (LC) locus to form the VDJ or VJ units encoding the HC or LC, respectively, of the resulting B cell receptor (BCR) (1, 2). B cells test these chains to ensure that the receptors are both functional and non-autoreactive. Thus, during early B cell development, B cells undergo several requisite checkpoints to ensure that a rearranged HC can properly pair with a rearranged LC and that the resulting HC : LC pair can potentially bind a foreign antigen but not self (3, 4). In eutherian mammals, after the HC locus rearranges (but before the LC loci rearrange, at the large pre-B cell stage of B cell development), a functional VDJ-rearranged HC is paired with a surrogate light chain (SLC, in complex with Igα and Igβ signaling molecules) to create a membrane-bound precursor BCR (pre-BCR) (5). Signaling by the pre-BCR signifies that the HC is capable of successful pairing with LC, which halts RAG expression and further HC rearrangement (6–8). The successful pairing of HC : SLC forms cross-linked pre-BCRs that enhance signaling and result in increased cell proliferation and subsequent expression of RAG for LC locus rearrangement (9–11). Only cells with HC that successfully pair with SLC undergo clonal expansion, enriching the population of B cells capable of forming functional BCR (9, 11). B cells then undergo negative selection to ensure receptors are self-tolerant; significantly self-reactive receptors are either altered *via* receptor editing or are eliminated from the population through apoptosis (5, 12). B cells with successful pre-BCR (functional yet non-autoreactive) then leave primary tissues (like bone marrow) as immature IgM^+^ B cells and migrate to spleen (or other secondary lymphoid tissues) where they mature and differentiate into long-lived mature follicular or marginal zone B cells. Upon activation, follicular B cells then differentiate into antigen-specific antibody-secreting plasma cells (13, 14). Similarly, thymocytes test rearranged β chains using a pre-T cell receptor alpha (PTCRA) surrogate to ensure functionality prior to rearranging the α chain (15, 16).

**Figure 1:**
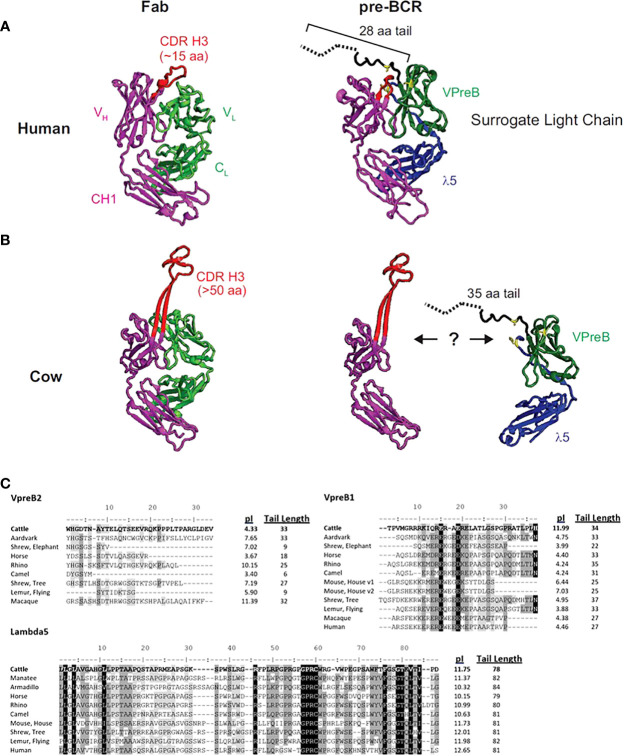
The human SLC may not accommodate an ultralong CDR H3. **(A)** A human fragment antigen-binding region (F_ab_; PDB 5ZMJ; doi: 10.2210/pdb5ZMJ/pdb) is shown next to a human pre-BCR composed of an immunoglobulin heavy chain (HC) paired with the surrogate light chain (SLC; PDB 2H3N; doi: 10.2210/pdb2H3N/pdb). **(B)** For comparison, a cow ultralong F_ab_ (PDB 5IJV; doi: 10.2210/pdb5IJV/pdb) is shown next to ultralong HC of that Fab with the human SLC from **(A)**. The structures are colored as follows: purple = HC, red = CDR H3, light green = light chain, dark green = VpreB1, blue = λ5, yellow = SLC residues that are important for interacting with the HC, dotted black line = part of VpreB1 tail that was truncated to obtain the crystal structure. Note that the VpreB1 tail may not engage the apex of the CDR H3 knob. **(C)** Amino acid alignment of VpreB1 (top), VpreB2 (middle), and IGLL1 tail regions, isoelectric point (pI), and amino acid tail length from eutherian mammal species. Though mice have two variants (v1 and v2) of VpreB1, neither mouse nor human have VpreB2. Note the high pI (more basic) tail of cattle VpreB1 and low pI (more acidic) tail of cattle VpreB2 compared to other species. Residues are shaded based on similarity using a Blosum62 scoring matrix (threshold = 1, gaps ignored; highlights indicate similarity: black = 100% similar; dark gray = 80 – 100% similar; light gray = 60 – 80% similar; white = <60% similar).

There are three known VpreB genes. VpreB1 and VpreB2 genes appear to be used interchangeably within the SLC, while VpreB3, which shares homology and nomenclature with VpreB1 and VpreB2, is instead thought to act as an Ig chaperone in the endoplasmic reticulum (17). The SLC is comprised of two invariant proteins: either VpreB1 or VpreB2 (together referred to as VpreB) and lambda5 (λ5) (18, 19). These proteins are structurally analogous to the V and C regions of a lambda LC (**Figure 1**). However, VpreB and λ5 differ from LC by the presence of unique tail regions (VpreB3, which is not part of the SLC, does not contain a substantial tail) (9, 20). VpreB contains a unique tail at the carboxy-terminus, termed the C-terminal unique region (C-UR), and λ5 (a homolog of the LC encoded by J_λ_-C_λ_ regions) contains a tail at the amino-terminus, termed the N-terminal unique region (N-UR). Together these tails form an analog of an unconnected third complementarity-determining region loop of a lambda LC (CDR L3) (9, 21), and these tails contact the CDR H3 extensively within the pre-BCR. The N-UR of λ5 forms the g-strand of the Ig-fold tertiary structure (typically encoded by the LC J segment) and contacts one side of the CDR H3, while the C-UR of VpreB forms a flexible probe that contacts the other side of CDR H3 and extends out from the antigen-binding site (9). A crystal structure of the human SLC paired with a HC F_ab_ fragment shows that the VpreB C-UR covers the CDR H3, thereby blocking the antigen binding site and preventing premature antigen binding (see **Figure 1A**). Several contact residues of SLC appear to demarcate a CDR H3 boundary, forming a “CDR H3 sensing site” that may control CDR H3 sequence selection, helping to shape the HC repertoire (9). VpreB aids selection for particular amino acids at specific locations at the center of antigen binding sites within CDR H3, thereby influencing antigen recognition and antibody production. For example, CDR H3 containing tyrosine residues at position 101 (Y101) pass pre-BCR checkpoints more often than those with residues other than tyrosine, and crystal structures of IgG Fab indicate that these Y101 residues often interact with antigen (22). Thus, an important regulator of early B cell development, the SLC plays a key role in generating an effective and highly diverse BCR repertoire for eutherian mammals.

The flexibility of the VpreB C-UR may allow it to interact with a diverse range of CDR H3 sequences and loop lengths. Since the VpreB tail interacts with the CDR H3 during pre-BCR testing, the length of the VpreB tail likely is vital to the roles of the SLC in shaping the HC repertoire and blocking access to antigen binding sites. In humans, CDR H3 are typically 8-16 amino acids long, and antigen binding sites (composed of three CDR each from HC and LC) form planar or undulating surfaces (23). However, CDR H3 loop lengths can vary widely between species. In cattle, CDR H3 loops are especially long, and ultralong (UL) antibodies (which range from 40-70 amino acids in length) form a distinct stalk and knob structure (23–25). For antibodies with longer CDR H3 or alternate structural forms, VpreB/λ5 tail flexibility alone may not be sufficient to bind the CDR H3 and influence selection. The actual length of a VpreB C-UR tail may be too short to extend over these elongated CDR H3 regions (**Figures 1B, C**). While it remains unclear whether UL antibodies pair with SLC during B cell development, if UL antibody repertoires are similarly shaped by interactions with SLC (as are canonical antibodies), then it is expected that species with elongated CDR H3 also should have extended VpreB C-UR tails.

Recently it has become clear that the structural diversity of antibody repertoires between vertebrate species can be dramatically different. In addition to the bovine UL repertoire, camelids and sharks have convergently evolved HC-only antibodies, and other species like chickens and rabbits have unusual disulfide-bonded structures within their CDR regions (25, 26). Furthermore, it has long been appreciated that different species have substantially different CDR H3 lengths (23, 27). The CDR H3 length and unusual antibody structures are features that likely are selectable during the SLC (pre-B) stage of B cell development. Therefore, understanding SLC evolution and its genetic and molecular features should allow greater understanding of the vertebrate antibody repertoire. We explored the origin of each SLC component (VpreB1, VpreB2, and IGLL1, which encodes the λ5 protein), as well as the related molecules VpreB3 and pre-T cell receptor alpha (PTCRA). We then assessed the relationship between sequence features of the SLC (i.e., length and charge) and HC (i.e., CDR H3 and DH length) within eutherian species. The results provide a framework for understanding antibody repertoires and their evolution across vertebrates.

## Methods

### Structural visualization

We downloaded crystal structures from the protein databank (PDB) (28) of a human Fab fragment [PDB: 5ZMJ (29)], a cow ultralong CDR H3 Fab fragment [PDB: 5IJV (30)], and the human SLC [PDB: 2H32 (9)]. We performed all structural visualization using PyMol software (PyMOL Molecular Graphics System, Version 1.2r3pre, Schrödinger, LLC).

### Sequence collection

We obtained all VpreB1, VpreB2, IGLL1, VpreB3, and pre-TCRα (PTCRA) gene sequences from one of four sources: National Center for Biotechnology Information (NCBI; ncbi.nlm.nih.gov), Ensembl Genome Browser (Ensembl; uswest.ensembl.org), UC Santa Cruz Genome Browser (UCSC; genome.ucsc.edu), or the BS Genome package in R. We began by searching the standard NCBI nucleotide database for orthologs of a gene. For unannotated sequences or those annotated with an alternate gene name, we used NCBI BLAST (31, 32) to search the nucleotide database using the mouse sequence as query and limiting the search to a single taxonomic identification number (tax id). When we did not obtain a hit, we used BLAST to search against the genome of a species using the RefSeq representative genome database and its tax id. As we compiled a sequence database, we used sequences from more closely related species as query within our BLAST search to refine our hits. In most cases, the query sequence matched a predicted gene location (annotated only with its location) within a chromosome or scaffold. In cases where hits did not match predicted genes, we downloaded the entire genomic region and regions overlapping the query sequence to identify gene exons. Additionally, we searched for conserved motifs and aligned sequences with genes from other organisms to validate exon and intron boundaries. Alternatively, when we identified an entire chromosome as a hit, we used the Table Browser at the UCSC Genome Browser to extract the desired region of the chromosome. Finally, we obtained the cattle VpreB1 sequence using the bosTau9 genome in the BS Genome package in R [also see (33)].

We considered VpreB1 sequences valid if they clearly possessed a unique C-terminal region tail, which often included 11-15 charged amino acids, and if they were 5’ proximal to the DNA topoisomerase III beta (TOP3B) gene. Similarly, we determined validity of VpreB2 sequences by the presence of a C-terminal region tail with 7-12 charged amino acids and its genomic location 5’ proximal to certain solute carrier family members (i.e., SLC5A4) near the lambda locus (IgL). Additionally, we searched for a conserved Glycine-Proline-Arginine-Cysteine (GPRC) motif within the tail region of IGLL1 sequences (approximately 30 amino acids 5’ of the lambda constant region). It should be noted that many genomes contained gene annotations for IGLL1 that do not encode the λ5 protein. Conversely, many IGLL1 genes were incorrectly annotated as IGLL3, IGLL5, IGLL7, or VpreB1 (see **Supplemental Table 1**). However, each sequence included in our database includes the GPRC motif and thus, likely encodes λ5 protein. In VpreB3, we searched for a conserved Valine-Phenylalanine-Proline-Glycine-Glutamine (VFPGQ) motif near the N-terminus of the sequence as well as the absence of an elongated C-terminal tail. **Supplemental Table 1** includes accession numbers or genomic locations for all gene sequences used in this study.

### Construction of tetrapod cladogram, sequence alignments, and phylogenetic trees

**Figure 2:**
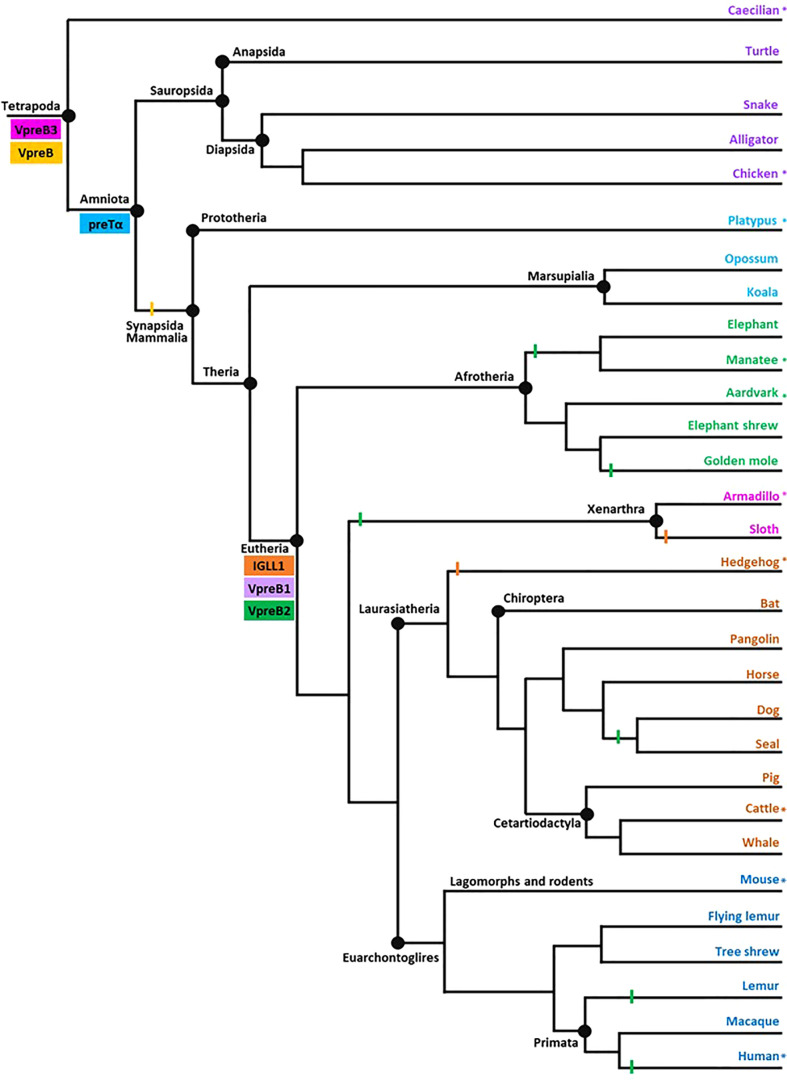
Model of surrogate light chain evolution in tetrapods. Cladogram of major tetrapod vertebrate groups illustrating the evolution of VpreB3, pre-T cell receptor alpha (PTCRA) chain, and genes used to construct surrogate light chain (VpreB1, VpreB2, and IGLL1). Multiple species inform each branch (see **Supplementary Tables 1**-**3** for accession numbers, amino acid sequences, and genomic locations of genes for included species). Colored rectangles indicate gene emergence for the gene named within the box. Similarly colored lines represent gene loss or gene absence (e.g., solid line at Mammalia; due either to gene loss or genome fragmentation) within that branch. Tree topology is based on mammalian phylogeny reported in Murphy et al. (34); individual branches that expand on this published tree are informed by published phylogenies of that group (35–38). Evolutionary distances are not to scale. Asterisks (*) next to a branch name indicates a species for which we examined genomes for synteny. Branch name colors coordinate groups within the cladogram to those of sequence alignments and phylogenies (see **Supplementary Figures 1**-**7**). Created with BioRender.com

We represented evolutionary relationships between tetrapods in a single cladogram based on the mammal phylogeny published in Murphy et al. (34). We used additional phylogenetic resources to refine or expand branches as necessary (35–38). We placed the origin of a gene at the earliest branch where we found that gene in at least two representative species. We then examined published genomes of organisms in a more basal branch to validate that the gene was not present below an origin point. We included at least one species from each tree branch (**Figure 2**) from which we found a SLC gene for our sequence analyses.

We performed all amino acid sequence alignments using the Clustal Omega (v. 1.2.2) alignment tool in Geneious Prime 2022.0.1 (https://www.geneious.com) using default parameters. We manually adjusted alignments as necessary and ordered sequences based on evolutionary relationships in our tetrapod cladogram (see **Figure 2**). For all alignments, species names were colored to coordinate with their placement in this phylogenetic tree, and residues within immunoglobulin domains (V region of VpreB1, VpreB2, and VpreB3; C region of IGLL1, and Ig region of PTCRA) were shaded based on similarity using a Blosum62 scoring matrix (threshold = 1, gaps ignored; highlights indicate similarity: black = 100% similar; dark gray = 80 – 100% similar; light gray = 60 – 80% similar; white = <60% similar). Within tail regions of VpreB1, VpreB2, IGLL1, and VpreB3, charged residues were highlighted blue (basic: arginine (R), pI=10.8; lysine (K), pI=9.8; histidine (H), pI=7.6) or red (acidic: glutamic acid (E), pI=3.2; aspartic acid (D), pI=3.0). **Supplemental Figures 1**-**5** depict sequence alignments for VpreB1 (55 species), VpreB2 (23 species), IGLL1 (39 species), VpreB3 (64 species), and PTCRA (63 species), respectively (see **Supplementary Table 2** for a list of amino acid sequences).

We determined phylogenetic relationships between sequences using the Geneious tree builder under default settings with a bootstrap resampling method of 2000 replicates. We coordinated branch colors of resulting trees with designated colors representing each SLC gene, as shown in **Figure 2** (VpreB1: purple; VpreB2: green; VpreB: yellow; IGLL1: orange; VpreB3: pink).

### Synteny analysis and annotation

Using the tetrapod cladogram (see above) to ensure balanced representation, we chose ten species for synteny analysis of SLC (VpreB1, VpreB2, IGLL1), VpreB3, and PTCRA genes based on their phylogenetic position within the major tetrapod radiations as well as the assembly state of their genome projects. We also assessed synteny within groups close to cattle to determine obvious differences in genomic structure that could inform our analyses of tail lengths in species that make ultralong CDR H3 antibodies, using horse (*Equus caballus*) and beluga whale (*Delphinapterus leucas*) as phylogenetically disparate bookends. We used published phylogenies (38, 39) to order species for synteny analysis among Bovidae. Finally, we examined gene synteny of glires (rabbits and rodents) to analyze differences in genomic placement of VpreB2 genes, using dog (*Canis lupus familiaris*) cattle (*Bos taurus*), and tree shrew (*Tupaia chinensis*) as flanking groups. **Supplemental Table 3** lists specific locations of each gene within assembled genomes of each species.

We ordered individual and/or blocks of orthologous genes to visually demonstrate conserved synteny between genomes of different tetrapod species. Thus, chromosomes and/or scaffolds may reflect either a forward (5’ to 3’) or reverse (3’ to 5’) orientation. Distances between genes do not necessarily correlate to genomic distances and often vary by genome. We anchored genomes for synteny analysis with genes for MIF (Macrophage Migration Inhibitory Factor, indicated by polygons labeled 1) at the 5’ end and TOP3B (DNA Topoisomerase III Beta, indicated by polygons labeled 12) at the 3’ end. We denote gene duplication with the number of duplicated genes below the gene polygon (e.g., two genes represented by “x2”). We represented blocks of syntenic genes or long unaligned regions between syntenic genes with the number of genes or the genomic distance between syntenic genes below the line, respectively. We indicated the location of the IgL when possible.

### Correlations between CDR H3 length, DH length, and VpreB tails

We obtained SLC gene sequences from cow (40); horse (41); rat, *Rattus norvegicus* (42); mouse, *Mus musculus* (43); human, *Homo sapiens* (44); rabbit, *Oryctolagus cuniculus* (45); little brown bat (*Myotis lucifugus*) and big brown bat, *Eptesicus fuscus* (46); dog (47); manatee, *Trichechus manatus latirostris* (48); and goat, *Capra hircus* (49). We then aligned sequences (as above) and counted residues within the CDR H3 for each species. The CDR H3 includes amino acid residues between the canonical cysteine at position 104 of the variable (V) region and the canonical phenylalanine or tryptophan at position 118 of the joining (J) region (thus, amino acids 105-117 of rearranged genes), based on unique numbering by the International Immunogenetics Information System (IMGT, www.imgt.org) (50, 51). We then calculated average and maximum CDR H3 lengths from these data. We obtained all germline DH sequences from IMGT except for goat DH, which were from (49). **Supplemental Table 4** list lengths of VpreB1, VpreB2, IGLL1, and VpreB3, and CDR H3 and germline DH.

For all correlation analyses, we used the Spearman correlation coefficient using the cor.test() function in R (https://www.R-project.org/). We created all plots using the ggplot2 package in R.

## Results

### Surrogate light chain genes emerged in eutherian mammal genomes

To begin to understand the features of surrogate light chains associated with repertoire evolution, we analyzed the tails of VpreB1 and VpreB2 of several species (**Figure 1**). Cows in particular have ultralong CDR H3 where the CDR H3 protrudes far from the typical antigen binding site and may not easily interact with a typical SLC (**Figure 1B**). The tail region of VpreB1 in cow was 34 amino acids, which was longer than human (27 aa) or mouse (25 aa) (**Figure 1C**). However, other species had similarly long tail regions including aardvark (33 aa, *Orycteropus afer*), rhino (35 aa, *Ceratotherium simum simum*), flying lemur (33 aa, *Galeopterus variegatus*), and tree shrew (38 aa). The tail length for VpreB1 in our dataset ranged from 16 amino acids (Natal long-fingered bat, *Miniopterus natalensis*) to 38 amino acids (tree shrew). A wider distribution of tail lengths was observed for VpreB2, which ranged from only six amino acids (camel, *Camelus dromedarius*) to 35 amino acids (beluga whale). We noticed VpreB tails contain several charged amino acids, so we calculated the predicted isoelectric point (pI) value of each sequence. Interestingly, comparing pIs of species that have both VpreB1 and VpreB2 genes, VpreB1 tails for most species were acidic, with nine out of 12 sequences being near pI ~ 4. A very notable exception was cow, which was very basic (pI = 11.99). VpreB1-2 from mice (further discussed below) was neutral at pI = 7.03. When the pI analysis was applied to VpreB2 tails, more variability was observed, and the pIs ranged from 3.40 (camel) to 11.39 (macaque, *Macaca fascicularis*). The pIs of tail sequences diverged significantly between VpreB1 and VpreB2 in only three species. In cow, the VpreB2 tail was acidic pI (4.33) whereas its VpreB1 was highly basic. The opposite was observed in both rhino and macaque, where VpreB1 tails were highly acidic (pI=4.24 and 4.38, respectively) and VpreB2 tails were highly basic (pI=10.15 and 11.39, respectively). Across species, length and pI fell within a narrower range for VpreB1 tails, while more diversity in length and charge were observed for VpreB2 tails. The major discrepancy in VpreB1 tail charge in cow may indicate a role in shaping its long CDR H3 repertoire.

**Figure 3:**
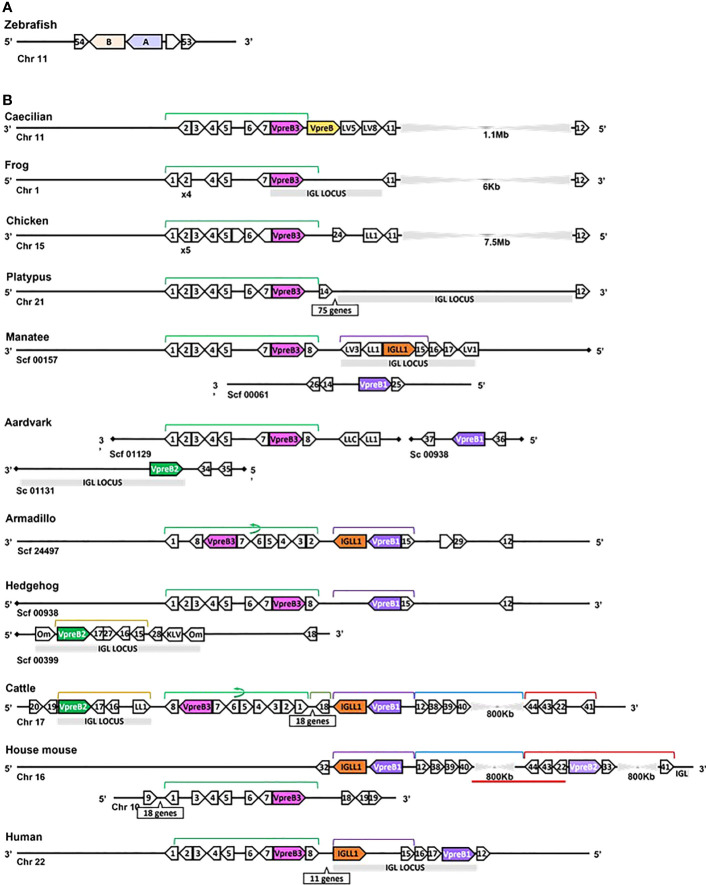
Locus synteny of VpreB3 and surrogate light chain (VpreB1, VpreB2, IGLL1) genes in tetrapod vertebrates. **(A)** Putative orthologs of both VpreB (**A**: *sid1*, light purple) and IGLL1 (light orange, **B**: *si:ch211-1a19.2*) in the zebrafish (*Danio rerio*) genome **(B)** In tetrapod vertebrates, relevant genes are shown in pink (VpreB3), purple (VpreB1), green (VpreB2), and orange (IGLL1). Polygons containing syntenic genes found in two or more species contain a number corresponding to the gene name. Polygons containing genes for uncharacterized proteins are empty. A solid line represents contiguous genomic sequence (chromosome or unplaced scaffold) and is denoted by the chromosome (Chr) or scaffold (Scf) to which it belongs. A line ending in a diamond shape indicates the end of a chromosome or scaffold and no further genes occur in that direction. Orientation of chromosomes or scaffolds is indicated by the 5’ or 3’ labels at the ends of the solid lines, and gene polygons point in their transcriptional direction. We indicate blocks of syntenic genes with the number of genes in a box below the line and long, unaligned regions with the genomic distance between syntenic genes. Colored lines above the polygons denote syntenic gene blocks, and lines containing an arrow indicate the syntenic block is inverted compared to caecilian. A red line below the polygons indicates an insertion of non-syntenic genes. The location of the lambda light chain locus (IGL, if present) is indicated by a gray rectangle. Orthologs between species are aligned vertically, but distances are not to scale. Polygons containing variable segments of lambda (LV) or kappa (KV) light chains and those containing lambda constant regions (LL) are reported with the annotated V or C segment number. In hedgehog, “Om” refers to the annotated gene *omega*, an alternate gene card name for VpreB2, that do not encode the VpreB2 protein. All other gene names are denoted by a number within the polygon (Polygon #: Gene name 1: *MIF*; 2: *SLC2A11*; 3: *DERL3*; 4: *SMARCB1*; 5: *MMP11*; 6: *CHCH210*; 7: *C22orf15*; 8: *ZNF70*; 9: *RGL4*; 10: *PDCH15*; 11: *CA15L*; 12: *TOP3B*; 13: *BABAM1*; 14: *OAS1*; 15: *PRAME*; 16: *ZNF280A*; 17: *ZNF280B*; 18: *SGLT1*; 19: *SLC5A4*; 20: *SLC5A1*; 21: *FDG4*; 22: *SLC25A1*; 23: *CLTCL1*; 24: *VPS29L*; 25: *WSCD2*; 26: *C12orf43*; 27: *RPL4*; 28: *PRAME12L*; 29: *PRAME6L*; 30: *ESS2*; 31: *GSC2*; 32: *SPAG61*; 33: *DGCR6*; 34: *ZNF596L*; 35: *ZNF596*; 36: *CDK20*; 37: *NUTM2D*; 38: *PPM1F*; 39: *MAPK1*; 40: *YPEL1*; 41: *HIRA*; 42: *PPIL2*; 43: *SPSPON*; 44: *THADA*; 45: *DGCR2*; 46: *DDX51*; 47: *SLC2A5*; 48: *SLC2A9*; 49: *TSSK2*; 50: *GALNT9*; 51: *DDT*; 52: *FAM200BIL*; 53: *WRD18*; 54: *ARRDC2*; see **Supplementary Table 3B** for gene names). Created with BioRender.com

**Figure 4:**
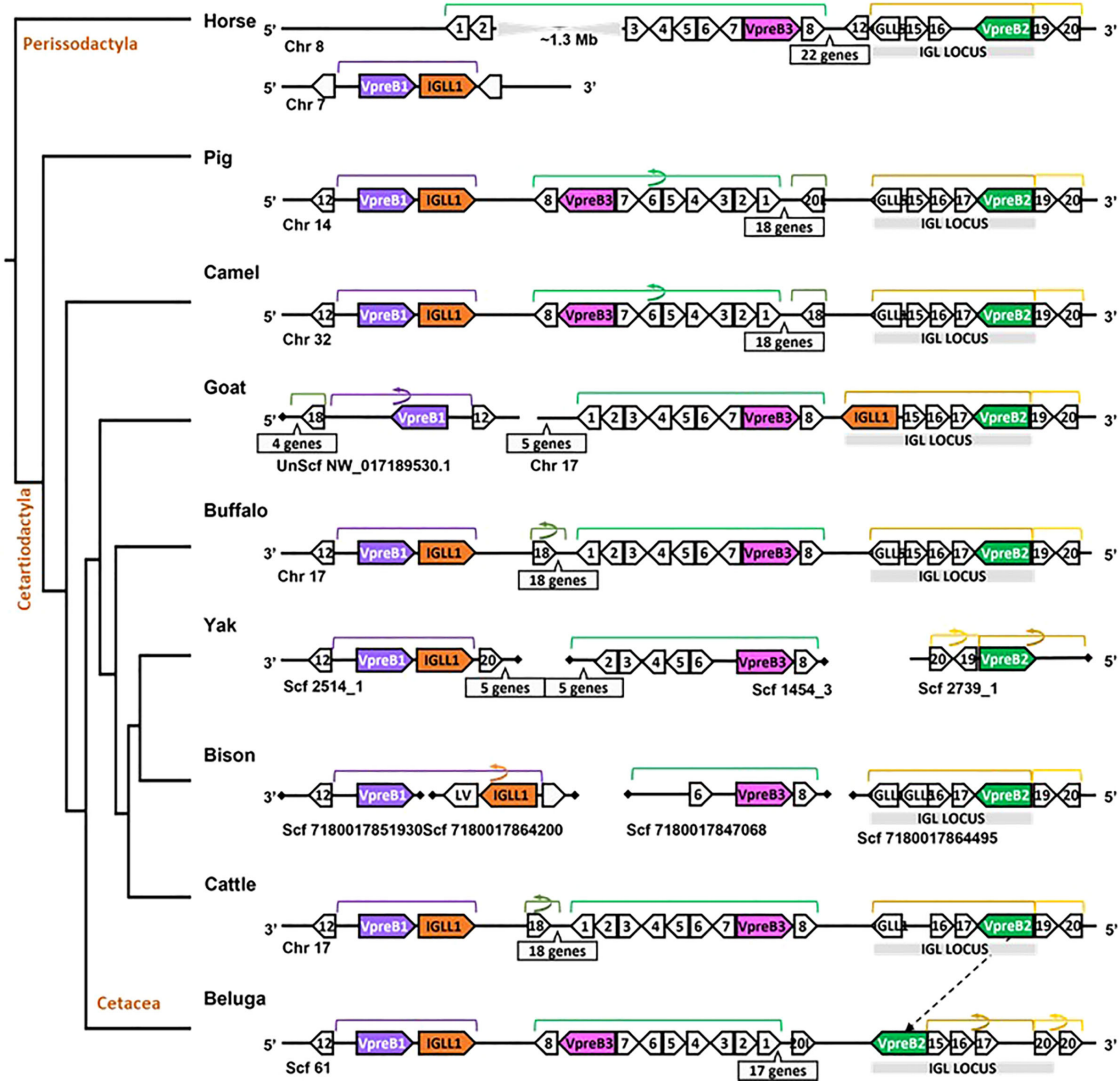
Locus synteny of VpreB3 and surrogate light chain (VpreB1, VpreB2, IGLL1) genes in cattle and their closest relatives. Genes are shown in pink (VpreB3), purple (VpreB1), green (VpreB2), and orange (IGLL1). Polygons containing syntenic genes found in two or more species contain a number corresponding to the gene name (see **Figure 3** for genes represented by each numbered polygon). Polygons containing genes for uncharacterized proteins are empty. A solid line represents contiguous genomic sequence (chromosome or unplaced scaffold) and is denoted by the chromosome (Chr) or scaffold (Scf) to which it belongs. A line ending in a diamond shape indicates the end of a chromosome or scaffold and no further genes occur in that direction. Blocks of syntenic genes are indicated with the number of genes in a box below the line and long, unaligned regions with the genomic distance between syntenic genes. Blocks of syntenic genes are indicated with the number of genes in a box below the line. Orientation of chromosomes or scaffolds is indicated by the 5’ or 3’ labels at the ends of the solid lines, and gene polygons point in their transcriptional direction. Colored lines above the polygons denote syntenic gene blocks, and lines containing an arrow indicate the syntenic block is inverted compared to horse. The location of the lambda light chain locus (IGL, if present) is indicated by a gray rectangle. An arrow between genomes of two species indicates a gene that is translocated in one species. Orthologs between species are aligned vertically, but distances are not to scale. See **Figure 1** and **Supplementary Table 3B** for a list gene names. A model cladogram is provided for reference. Tree topology is informed by published phylogenies (38). Created with BioRender.com

After the initial evaluation of several SLC genes, we took a more comprehensive effort to characterize the genetics, evolution, and molecular features of SLC components. Through this effort we found genes for VpreB1 (n=57 species), IGLL1 (n=39 species), and VpreB2 (n=24 species) in genomes of eutherian mammals only, suggesting all three genes originated together at the divergence of eutherian mammals from marsupials (**Figure 2**; **Supplementary Figures 1, 2A, 3-5**). VpreB2 did not occur in genomes of all eutherian mammals, and its absence from a genome was not predictable by clade branch. The absence of VpreB2 in some Afrotherian (*e.g.*, elephants, *Loxodonta africana* and manatees) and Xenarthran mammals may stem from incomplete or poorly assembled genomes (see **Figure 3**). However, VpreB2 was noticeably absent from genomes of humans and non-human primates (except monkeys) and most carnivores, cetaceans, and bats. Highly scaffolded genome assemblies within the regions containing VpreB1, VpreB2, and IGLL1 made syntenic conservation of genes difficult to assess. For example, we were unable to locate IGLL1 within sloth (*Choloepus didactylus*) or hedgehog (*Erinaceus europaeus*) genomes, despite its presence in genomes of all other eutherian mammals examined. Of note, we did identify the IGLL1 gene in pigs (*Sus scrofa*), despite published reports indicating its absence from the pig genome (52). As in other species, IGLL1 in pigs flanked VpreB1 just 5’of VpreB3 (see **Figure 4**).

While we typically located VpreB1 and IGLL1 close together and near the TOP3B gene, the position of VpreB2 was less conserved. In most cases, IGLL1 was adjacent to or within a few genes of VpreB1 while VpreB2 was located by itself within IgL (see **Figures 3**, **4**). In humans (which do not have VpreB2), IGLL1 and VpreB1 were separated by three syntenic genes (PRAME (polygon 15), ZNF280A (polygon 16), and ZNF280B (polygon 17)) and demarked the beginning and end of IgL. These same three genes occur 5’ of IGLL1 in manatee, but VpreB1 appeared on a separate scaffold from IGLL1, and two additional genes (oas1 and c12orf43) occur 3’ of VpreB1, revealing a slightly altered gene order. In mice, all three genes were found on chromosome 16 about 1Mb upstream (5’) from IgL. A similar translocation occurred in horse, whereby IGLL1 and VpreB1 genes translocated to chromosome 7, leaving VpreB2 within IgL downstream (3’) from VpreB3 (**Figure 4**). In Cetartiodactylans (except goat) syntenic order was mostly conserved, with VpreB1 and IGLL1 upstream of VpreB3 and VpreB2 located within IgL downstream of VpreB3. In goat, we located IGLL1 within the lambda locus.

**Figure 5:**
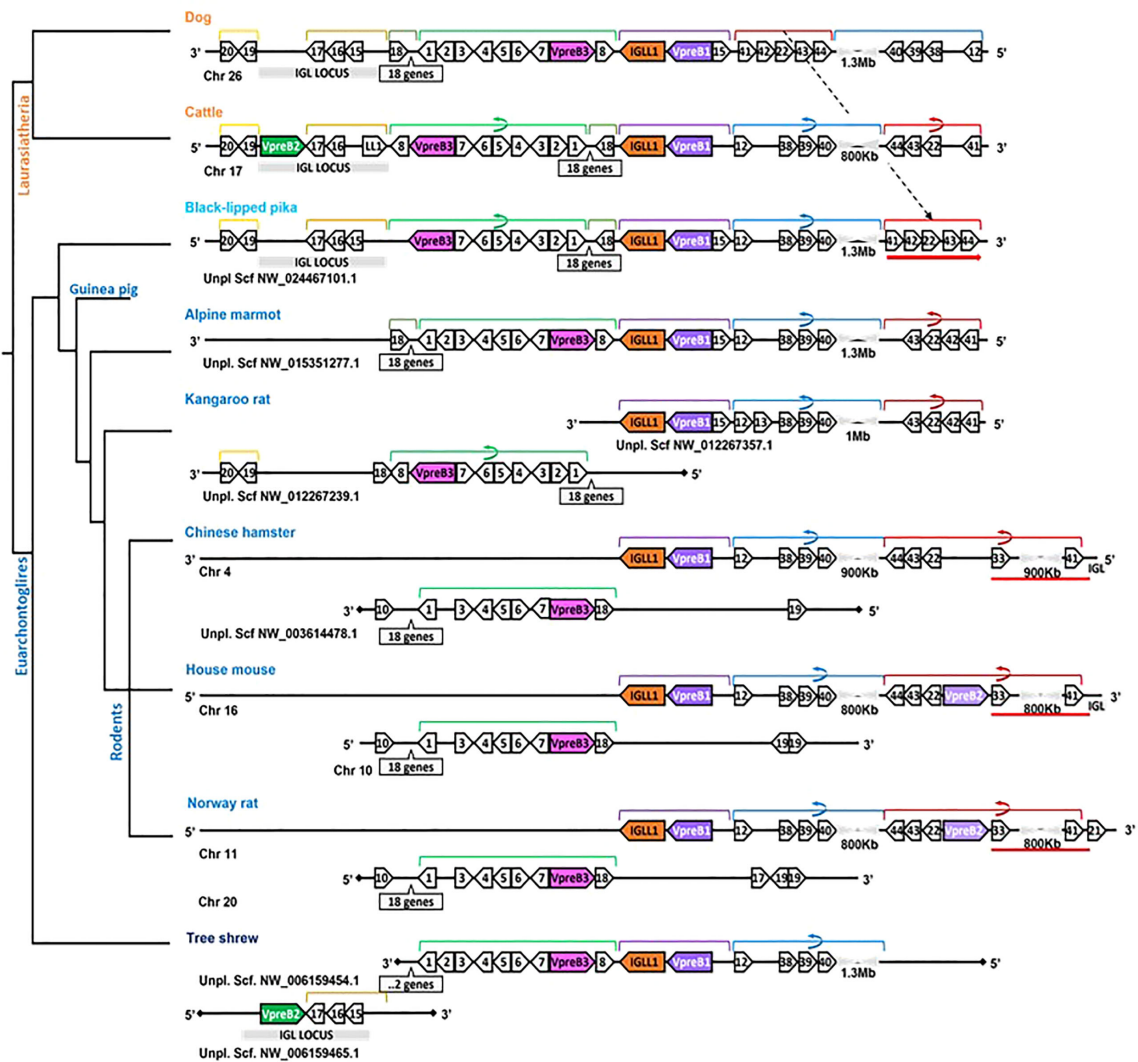
Locus synteny of VpreB3 and surrogate light chain (VpreB1, VpreB2, IGLL1) genes in Glires (rabbits and rodents) and their closest relatives. Genes are shown in pink (VpreB3), purple (VpreB1), green (VpreB2), and orange (IGLL1). Polygons containing syntenic genes found in two or more species contain a number corresponding to the gene name (see Figure 3 for gene names represented by each numbered polygon). Polygons containing genes for uncharacterized proteins are empty. A solid line represents contiguous genomic sequence (chromosome or unplaced scaffold) and is denoted by the chromosome (Chr) or scaffold (Scf) to which it belongs. A line ending in a diamond shape indicates the end of a chromosome or scaffold and no further genes occur in that direction. Orientation of chromosomes or scaffolds is indicated by the 5’ or 3’ labels at the ends of the solid lines, and gene polygons point in their transcriptional direction. Colored lines above the polygons denote syntenic gene blocks, and lines containing an arrow indicate the syntenic block is inverted compared to dog. A red line below the polygons indicates an insertion of non-syntenic genes that includes this second VpreB gene in Glires. The location of the lambda light chain locus (IGL, if present) is indicated by a gray rectangle. Orthologs between species are aligned vertically, but distances are not to scale. A model cladogram is provided for reference. Tree topology is informed by published phylogenies (35–38). Created with BioRender.com

In general, genomic organization of the SLC genes in mammals appeared less conserved than VpreB3 or PTCRA genes. For example, in armadillo (*Dasypus novemcinctus*), hedgehog, cattle, and mouse, VpreB1 was flanked 5’ by TOP3B and 3’ by IGLL1 (if present) and a syntenic block of genes that contains VpreB3, and (except in mice) VpreB2 was located within IgL (**Figure 3**). In rodents, VpreB2 was located between SLC25a1 (polygon 22) and HIRA (polygon 41) within an inverted block of syntenic genes quite distal from VpreB1 and IGLL1 (>800kb upstream) (see **Figure 5**). The genomic location of rabbit VpreB2 was unclear within the current genomic assembly. For most species that have both VpreB1 and VpreB2 genes, these two genes were distinct from one another (with VpreB1 sharing around 50% amino acid identity with VpreB2). However, in rabbits and rodents, VpreB2 shared at least 95% of residues with VpreB1 (>97% nucleotide and amino acid identity; **Supplementary Figure 2B**). Consequently, while VpreB1 and VpreB2 sequences from other species branch into separate clades, both genes from glires grouped together within the VpreB1 clade, consistent with the idea that the VpreB2 gene in glires arose independently from the VpreB2 of other mammals (**Supplementary Figures 6**, **7**). Sequence conservation between glire VpreB1 and VpreB2 genes was reflected in the charge observed in VpreB tails, with isoelectric point nearly identical in glire genes but vastly different in other species (i.e., cattle VpreB1: pI =11.99; VpreB2: pI=4.33; see **Figure 1C**), likely indicating less neofunctionalization of the two genes in glires. We suggest that VpreB genes in glires be corrected to reflect a more recent duplication event, with gene names changed to VpreB1-1 and VpreB1-2. This change would clearly discriminate the distinct VpreB2 genes of other species from those of glires.

Phylogenetic analyses of VpreB1, VpreB2, and VpreB3 sequences indicated that VpreB3 originated first in the genome, with VpreB1 diverging most recently (see **Supplementary Figure 6**), supporting gene origin placements on our cladogram (**Figure 2**). VpreB1 sequences clustered together in a single branch within VpreB2 (94% consensus support) (see **Supplementary Figures 6**, **7**). VpreB2 and VpreB1 genes from the same species (except glires, see above) generally shared fewer than 62% of residues. IGLL1 sequences were conserved with eutherian mammals, especially within constant regions (greater than 70% similarity between species compared to less than 60% similarity in tails between groups; **Supplementary Figure 3**). As expected, sequence identity of all three SLC genes was high among species within the same order (e.g., Carnivora, Artiodactyla, or Primata) but showed little conservation between even closely related groups. For example, IGLL1 sequences from rodents alone shared 72 – 98% amino acid identity, but sequences from rodents shared only 52 – 64% amino acid identity with sequences from primates (**Supplementary Figure 3**). IGLL1 N-terminal tail regions were less conserved than constant regions (C), though IGLL1 C shared only 55 – 70% amino acid identity with groups outside their evolutionary branch. VpreB1 and VpreB2 showed similar homology trends, though identity generally was less conserved between evolutionary groups (VpreB1: 37 – 74% amino acid identity; VpreB2: 22 – 60% identity). Further, VpreB1 genes were more conserved (>51% amino acid identity) overall than those of VpreB2 (>32%). Tail regions were conserved only among members of an evolutionary order. When aligned together, VpreB1 and VpreB2 group into separate phylogenetic branches, except in rodents, as noted above.

In addition to VpreB3, we identified a second VpreB gene in the caecilian (*Rhinatrema bivittatum*genomes) genome. Annotated as Ig Iota chain (a GeneCard alias for VpreB1) in the caecilian genome, this sequence was flanked 3’ by VpreB3 and 5’ by at least two LC variable segment genes (**Figure 3**). Phylogenetically, this second VpreB gene clustered with VpreB2 (and VpreB1; **Supplementary Figures 6**, **7**). Additionally, we found transcripts from alligator (*Alligator mississippiensis*) and turtle (*Chelonoidis abingdonii*) that aligned more closely with this second caecilian gene than to VpreB3. Alignments further illustrated this divergent VpreB lineage, with longer tail regions incorporating numerous polar residues indicative of VpreB1 and VpreB2 but not VpreB3 (**Supplementary Figure 8**). These results indicated this new gene was a distinct lineage from VpreB3 and suggests it could be a precursor to VpreB2 that was lost in non-eutherian mammals.

Since we were specifically interested in features of VpreB and IGLL1 from species with unusually long CDR H3 regions, we conducted a separate in-depth analysis of genomes from cattle and their nearest evolutionary neighbors (**Figure 4**). We observed greater conservation in genomic organization of SLC genes than in all eutherian mammals, with only minor exceptions. Like mouse, SLC genes of horse (one branch below Artiodactyla) occurred on two separate chromosomes, with VpreB2 and VpreB3 on chromosome 8 and VpreB1 and IGLL1 on chromosome 7. In all genomes except beluga whale (one branch up from Artiodactyla), we found VpreB2 at the 5’ end of IgL, flanked 5’ by two solute carrier family member genes (polygon 19, SLC5A1, and polygon 20, SLC5A4) and 3’ by two zinc finger protein genes polygon 16, ZNF280A and polygon 17, ZNF280B). In beluga whale, the VpreB2 gene translocated to the 3’ end of IgL but in the same transcriptional orientation. Additionally, there was an inverted block containing 26 syntenic genes including VpreB3 in pig, camel, and beluga whale compared to cattle, yak, buffalo, and likely bison (see **Figure 4**, polygons labeled 1 to 8, VpreB3, and 17 additional genes in pig, camel, buffalo, yak (*Bos mutus*), cattle, and beluga). This gene block may also occur in goat, though the middle seven genes are missing from both chromosome 17 and scaffold NW_017189530.1. Genes for VpreB1 and IGLL1 typically are adjacent to each other upstream of VpreB3, typically flanked by TOP3B at the 3’ end of VpreB1. However, the IGLL1 gene in goat was downstream from VpreB2. Thus, within Artiodactylans, genomic organization of SLC genes was mostly conserved.

### Species with ultralong CDR H3 also have longer SLC tail regions

**Figure 6:**
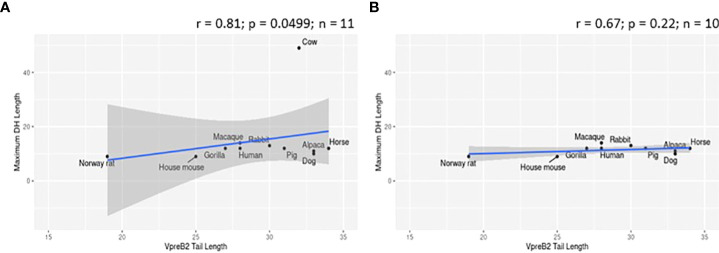
VpreB2 tails are longer in species with elongated DH gene segments. **(A)** VpreB2 tail lengths are highly correlated with maximum DH length (r= 0.81; p=0.0499; n = 6). **(B)** However, this relationship disappears when cattle are removed from the dataset (r=-0.029; p=0.957; n = 5).

To evaluate whether elongated CDR H3 regions co-evolved with elongated VpreB tails, we evaluated SLC genes from 64 species across many vertebrate orders. Of the 64 species studied, 57 had VpreB1 sequences and 39 had IGLL1 sequences, whereas only 24 had VpreB2. We predicted that, if the C-terminal tails of VpreB1 or VpreB2 need to interact with CDR H3, then species with longer, protruding CDR H3 would require longer VpreB1 and VpreB2 tails. To test this idea, we compared VpreB tail length to average and maximum CDR H3 length and to average and maximum DH length (since the DH encodes the majority of CDR H3). While CDR H3 length (either average or maximum) did not correlate significantly with either VpreB1 or VpreB2 tail length (p > 0.2 for all analyses), the relationship between CDR H3 length and VpreB1 tail length (negative: r = -0.18 and r = -0.35 for average and maximum CDR H3 length, respectively) was opposite that of VpreB2 tail length (positive: r = 0.53 and r = 0.27, respectively). However, species with longer maximum DH lengths also had longer VpreB2 tail lengths (r = 0.81, p = 0.0499; n = 6), though the ultralong cattle DH strongly influenced this relationship (**Figure 6**). It is worth noting that these analyses were limited by the lack of published immunogenetic data for most species in our database. Of the 64 species in our database, we could calculate average CDR H3 for only 14 species and average DH for only 11 species, and we had complete information (VpreB1, VpreB2, CDR H3, and DH) for only seven species (rabbit, Norway rat, house mouse, goat, horse, cow, and macaque), three of which are glires.

**Figure 7:**
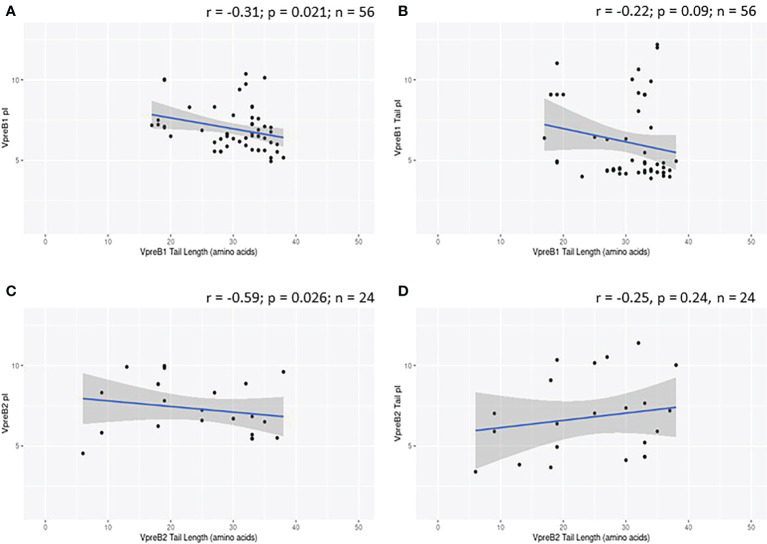
VpreB tail length is negatively correlated with isoelectric point (pI) of tail residues. **(A)** pI and length of VpreB1 tail regions show a weak negative correlation (r = -0.31; p = 0.021; n = 56), though **(B)** this relationship is not due to pI of VpreB1 tail residues (r = -0.22; p = 0.09; n = 56). **(C)** pI and length of VpreB2 tail regions show a moderate negative correlation (r = -0.59; p = 0.026; n = 24), and this **(D)** relationship was unrelated to pI of VpreB2 tail residues (r = -0.25; p = 0.24; n =24).

**Figure 8:**
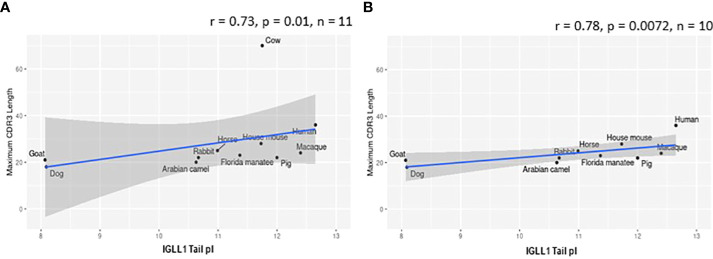
Isoelectric point (pI) of IGLL1 tails increases with increasing CDR H3 length. **(A)** pI of IGLL1 tails was highly correlated with maximum CDR H3 length (r= 0.73; p=0.01; n = 11). **(B)** This relationship remains even when cattle (with ultralong DH segments) are removed from the dataset (r=-0.78; p=0.0072; n = 10).

While evaluating VpreB1 and VpreB2 sequences, we observed that their tail regions contained many charged amino acids (**Figure 1C** and **Supplementary Figure 1**, **2A**). This led us to consider whether tail length could affect the predicted isoelectric point of VpreB1 and VpreB2. Indeed, the pI of the entire VpreB1 protein decreased as VpreB1 tail length increased, suggesting that the protein became more acidic as the tail got longer (r = -0.31, p = 0.021, n = 56; **Figure 7A**). Although the VpreB1 tail contained many acidic and basic residues, the tail did not contribute significantly to this relationship (r = -0.22, p = 0.09, n = 56; **Figure 7B**). We observed the same trends with VpreB2 (VpreB2 pI: r = -0.59, p = 0.026, n = 24; VpreB2 tail pI: r = -0.25, p = 0.24, n = 24; **Figure 7C, D**). Unexpectedly, we found that pI of IGLL1 tails increased significantly (becoming more basic) as maximum CDR H3 increased (r = 0.73, p = 001, n = 11; **Figures 1C**, **8**). Thus, as CDR H3 length increased, the IGLL1 tail became more positively charged (basic), and as VpreB tail length increased, the V segment (untailed) portion of the VpreB protein that interacts with the IGLL1 tail became more negatively charged (acidic). These adjustments in charge as CDR H3 length got longer suggest a role for the SLC in interacting with additional charged residues in the CDR H3, which may be structural (e.g., holding the CDR in place), functional (e.g., shaping the repertoire through selection), or both.

### VpreB3 is found in all tetrapod vertebrates while PTCRA arose in amniotes

We found orthologs of VpreB3 genes in all tetrapod groups (n=64 species) and orthologs of PTCRA in all groups except amphibians (n=63 species) (**Figures 2**, **3**; **Supplementary Figures 4**, **5**, **9**, **10**). VpreB3 was flanked 5’ by *C22orf15* (chromosome 22 open reading frame 15; polygon 7) in all tetrapod genomes except chicken, in which it was flanked by an uncharacterized protein (LOC769646; unlabeled polygon) oriented in the same direction as *C22orf15* in other species (see **Figure 3**). In all eutherian mammals except mouse, VpreB3 was flanked 3’ by *ZNF70* (zinc-finger protein 70; polygon 8) and at least one downstream lambda gene (or IgL). However, in mice, VpreB3 (on chromosome 10) was flanked 3’ by *SLC5A2* (solute carrier family 5 member 2; polygon K), while a downstream block of syntenic genes (including IgL) translocated to chromosome 16. In armadillo, VpreB3 was located within an eight gene inverted syntenic block but was still located 5’ of lambda (**Figure 3**). Pig, camel, and beluga whale genomes contain this same inversion, though notably this inverted syntenic block contains 18 additional genes 3’ of *MIF* (macrophage migration inhibitory factor; **Figure 4**).

In caecilians, VpreB3 was flanked 3’ by a second VpreB gene and two downstream lambda V genes (*IGLV5*, *IGLV8*) on chromosome 11 (**Figure 3**; **Supplementary Figure 9**). We found this second VpreB gene in turtle, alligator, and snake, and these genes shared greater identity to VpreB2 of eutherian mammals than to VpreB3 of all species (**Supplementary Figures 6**, **7**). The frog VpreB3 gene was located on chromosome 1 within IgL, and the chicken VpreB3 was flanked 3’ by *VPS29L* (a vacuolar protein sorting gene; polygon B) and a lambda C gene on chromosome 15. VpreB3 was found on platypus chromosome 21 flanked 3’ by *OAS1* (oligoadenylate synthase) gene (polygon 14), with IgL downstream but separated by a large, unaligned region containing 75 genes (**Figure 3**). Amino acid alignments of VpreB3 sequences suggest VpreB3 was conserved within tetrapods, with human VpreB3 sharing 63% of residues with caecilian VpreB3 (**Supplementary Figure 4**).

All amniotic tetrapod genomes contained a PTCRA gene (**Supplementary Figures 5**, **10**). In mammals, PTCRA was flanked 5’ by *RPL7L1* (ribosomal protein L7-like 1) and 3’ by *CNPY3* (canopy FGF signaling regulator 3). In chicken (*Gallus gallus*), PTCRA was flanked 5’ by *POLR1B* (RNA polymerase I subunit B) rather than *RPL7L1* and 3’ by *CNPY3*. We searched the *Xenopus* genome 5’ of the *CNPY3* gene but did not locate PTCRA (**Supplementary Figure 10**). PTCRA sequences share the greatest similarity within a cladistic branch (e.g., Euarchontoglires), primarily due to the considerable sequence variation within cytoplasmic tail (Ct) regions (see **Supplementary Figure 5**). However, alignments with Ct regions removed from sequences indicate a high degree of similarity within immunoglobulin (Ig), connecting peptide (Cp), and transmembrane (Tm) domains, with human PTCRA genes sharing more than 80% of residues with other eutherian mammals, 74% of residues with marsupial mammals, 67% of residues with monotreme mammals, and at least 49% of residues with non-mammal tetrapods (data not shown). All sequences incorporated the two cysteines essential for making an intrachain disulfide bridge (positions 28 and 88 in our alignment) and the tryptophan required for stabilizing the Ig-fold tertiary structure (position 43). Thus, PTCRA was highly conserved across tetrapod species.

## Discussion

The surrogate light chain provides a quality control checkpoint for immunoglobulin heavy chains during development, and importantly, also plays a role in shaping the antibody repertoire through its CDR H3 interactions (9). With the discovery of several unusual antibody structural and genetic features in different species, for example HC-only antibodies in camelids and ultralong CDR H3 antibodies in cows, an understanding of HC-SLC molecular and genetic features in these extreme circumstances may shed light on fundamental mechanisms of repertoire development. CDR H3 length, in particular, is known to impact viral neutralization, with rare long CDR H3s being important in broadly neutralizing anti-HIV antibodies (53, 54). Interestingly, circulating human B cells with pre-BCRs were found to have unusually long, self-reactive, CDR H3 regions (55). Thus, understanding B cell development in the context of HC-SLC interactions has medical implications. The antibody repertoire is shaped by the genome-encoded sequences of its V, D, and J gene segments, their relative usages, junctional diversity factors including terminal deoxynucleotidyl transferase (TdT), and somatic hypermutation mechanisms. The selection step utilizing the SLC is also an important process impacting the expressed antibody repertoire in any given species. In this regard, features of the SLC (including tail lengths, pI, or other molecular properties) or even the usage of alternative SLC components, like multiple VpreB genes, has the potential to significantly alter the repertoire. Therefore, we analyzed several features of SLC components, including (i) the number of VpreB genes, (ii) evolutionary relationships between SLC components, (iii) molecular features of tail lengths, and (iv) correlations between SLC features and repertoire features like CDR H3 length.

### SLC genes evolved in eutherian mammals whereas PTCRA evolved in amniote tetrapods

The B and T cell-based adaptive immune system found in mice and humans also occurs in cartilaginous fish. However, certain components of B and T cell development deemed crucial to maintaining the efficiency and effectiveness of this system in mice and humans did not evolve at the same time. We examined the evolutionary origins of SLC and PTCRA components, genes encoding surrogate chains for immunoglobulin light chain (LC) and T cell receptor alpha chain (TCRα), respectively. PTCRA first emerged in amniote tetrapods as an invariant, non-rearranging surrogate chain that partners with TCR beta chain (TCRβ) and CD3 signaling complex, forming a pre-TCR on the immature (DN) T cell surface (56). PTCRA plays no known role in development of γδ T cells (57). The invariant VpreB and IGLL1 proteins then arose in eutherian mammals as components of a LC surrogate that combines with HC and Igα/Igβ signaling molecules to form a pre-BCR on immature B cells (8, 9). These surrogate chains are considered crucial to B and T cell development, primarily by linking HC or TCRβ to signaling proteins within the BCR or TCR complex, facilitating signaling through the pre-BCR or pre-TCR on the surface of pre-B or pre-T cells, respectively (8, 15, 17, 56). Because lymphocyte receptor chains are sequentially rearranged during development, this signaling permits pre-B or pre-T cells to test rearrangements of the primary chain (HC or TCRβ, respectively) at the cell surface prior to rearranging the secondary chain (LC or TCRα, respectively), ensuring the chain is functional (8, 17, 58).

In mice and humans, disruptions to pre-BCR or pre-TCR components impair the ability of immature B or T cells to transition into mature cells (8, 9, 56, 58). For example in B cells, the pairing of HC with a SLC stabilizes the pre-BCR complex and confirms the rearranged HC can properly fold and assemble into a functional receptor, thus predicting whether the chain will be able to pair successfully with LC (59). Cell division at this stage also allows a single functional HC to pair with multiple alternative LCs, thus efficiently expanding the repertoire (59). Signaling by successful HC : SLC or TCRβ: PTCRA pairings results in cessation of primary chain locus rearrangement and induces clonal expansion, creating numerous cells containing replicas of the successful chain (9, 56, 60). Only then do cells begin rearranging a LC or TCRα locus. Thus, this sequential rearrangement strategy safeguards against production of nonfunctional receptors, a critical quality control mechanism for proper immune system function (59).

Smelty et al. (56) first described the evolution of PTCRA within amniotes, providing a detailed synteny of genes around PTCRA within the genomes of nine amniote species. We confirmed these findings, identifying a PTCRA gene with a similarly conserved gene synteny in genomes of all amniote tetrapod groups [*CNPY3* was downstream from *PTCRA* in all tetrapods and *POLR1B* upstream in all mammals (**Figure 5**)]. While updated chromosome assemblies have since altered the published gene organization along chromosome 5 in *Xenopus laevis* [see **Figure 1** of (56)], we still were unable to locate PTCRA in this species. With our expanded species database, we confirmed amino acid sequence conservation within mammals, with most sequences containing conserved cysteines important for creating the intrachain disulfide bridge (C28 and C88 in our alignment), the tryptophan (W43) essential for stabilizing tertiary structure of the Ig fold, and residues important for correct folding of the protein (see **Supplementary Figure 5**) (56). Further, we found that cytoplasmic tail regions in sequences from non-eutherian mammals were shorter and contained fewer (and in some cases no) proline residues, which in mice and humans are important for pre-TCR signaling and T cell development (**Supplementary Figure 5**) (61, 62). This suggests that T cells of early tetrapods (and likely non-tetrapod jawed vertebrates) may use altered approaches to ensure functionality of their TCR, perhaps employing strategies like somatic hypermutation to further diversify receptors during T cell development in the thymus (as observed in nurse sharks) (63, 64).

With the advent of a TCR quality control system, eutherian mammals then acquired the ability to similarly test BCR through the evolution of genes comprising the SLC (VpreB and IGLL1), as was suggested by early work in marsupials (65). VpreB and IGLL1 are homologous to the V region and J_λ_-C_λ_ region of LC, respectively, and associate with Igα and Igβ signaling molecules and HC to form the pre-BCR complex (8, 17). VpreB and IGLL1 each also encode a unique region (UR) containing many charged residues. The VpreB-UR (C-UR, found at the carboxy terminus of the protein) contains many negatively charged residues, while the λ5-UR (N-UR, found at the amino terminus of the protein) contains many positively charged residues (see **Supplementary Figures 1**, **3**). These URs project out from the SLC complex and are essential for stabilization and folding of the pre-BCR structure (8). Additionally, the F_ab_-like arms of one pre-BCR interact with the F_ab_-like arms of other pre-BCR, creating a chain of pre-BCRs on the cell surface. These cross-linked pre-BCR “rafts” bring HC into proximity of Igα/Igβ to induce intracellular signaling (9, 66). While neither SLC nor PTCRA signal allelic exclusion at the respective HC or TCRβ locus (8, 58), both pre-BCR and pre-TCR are essential for αβT cells and B cells to develop properly, as signaling by the pre-BCR or pre-TCR induces proliferative expansion of pre-B cells or double-negative (DN) T cells, respectively (8, 57, 58). Pre-B cells can develop to the small B cell stage without undergoing cell proliferation, but these cells also produce far fewer immature B cells (8). Similarly, pre-T cells can develop into single-positive (SP) thymocytes in absence of proliferation but with very low efficiency, generating substantially fewer mature thymocytes (47, 48). Thus, for eutherian mammals (especially mouse and human), generation of sufficient numbers of B and T cells in the immune repertoire depends on these surrogate chains.

We located VpreB1 genes in all eutherian genomes examined and IGLL1 in all genomes except sloths and hedgehogs, though we suspect IGLL1 will be found in these species as genome assemblies improve (**Figures 2**, **3**). Phylogenetic analyses of VpreB indicate VpreB1 sequences are highly conserved, with at least 74% sequence similarity between VpreB1 of Afrotherians and primates (**Supplementary Figure 1**). IGLL1 sequences show similar conservation (greater than 70% sequence similarity among eutherian IGLL1 sequences; **Supplementary Figure 3**). While arising at the same time as VpreB1 and IGLL1, VpreB2 does not occur in all eutherian groups, being notably absent from many Afrotherians (elephant, manatee, and golden mole) and all Xenarthrans (armadillo and sloth), carnivores, and primates (except monkeys) (**Figure 2**; **Supplementary Figure 2**). Compared to VpreB1, VpreB2 sequences are less conserved (only 60% similarity between Afrotherian and primate sequences) and contain fewer polar residues within their UR tails (23% of total residues in VpreB2 versus 40% in VpreB1; **Supplementary Figures 2**). These differences suggest these two genes likely form pre-BCR with slightly different functions.

VpreB3 is not a component of the SLC in B cells, though its highly conserved presence in all tetrapods suggests it plays a primordial role in B cell development. While the precise function of VpreB3 still is relatively unknown, early work in pre-B cell lines suggests it associates intracellularly with nascent Igµ chains early in B cell development and typically does not leave the endoplasmic reticulum (ER) (17, 67). In chickens, VpreB3 binds free LC and prevents it from leaving the ER, thereby regulating LC maturation and secretion (67). HC typically must be assembled with LC for secretion, but LC can be secreted alone (“free LC”) (59). Unchecked LC secretion can lead to pathological complications (*e.g.*, LC-induced mast cell activation, progression of autoimmune disease, and precipitation in heart, lung, kidney, or liver tissues) (67). Thus, VpreB3 may have an important regulatory role in the adaptive immune system. Further, while VpreB3 is primarily found in bone marrow and germinal centers of lymphoid tissue, VpreB3 also is expressed by Purkinje cells (cerebellum) and adrenocortical cells and may function in intracellular calcium regulation during aldosterone biosynthesis within the adrenal cortex (68). These functions suggest that VpreB3 may interact with components necessary for repertoire development in B cells but have other chaperone roles in other cell types. Whether VpreB3 plays any specific role in BCR development, especially in non-eutherian mammals without SLC, currently is unknown.

The majority of what we know about how B and T cells develop their receptor repertoires derives from humans and mouse models. Thus, B cells sequentially rearrange a HC first, requiring a surrogate as a LC proxy for testing functionality of the rearranged HC. Because this mechanism is so important in mice and humans for producing functional receptors and shaping receptor repertoires, it is imperative to interrogate whether non-eutherians (especially non-tetrapods) use this same strategy or have alternate mechanisms to ensure receptor functionality. Pigs employ an alternate strategy for receptor formation in which B cells first recombine Ig LC gene segments, forming authentic LC rearrangements *before* beginning to rearrange HC (69, 70). Further, cells recombine Ig kappa (Igκ) LC gene segments first and (because there are no recombined HC) then replace the majority of these Igκ LC with rearranged Igλ LC (70–72). Once rearranged, HC procure one of these rearranged *authentic* Ig LC for surface expression and functional testing rather than employing a surrogate, despite containing both (presumably functional) VpreB1 and IGLL1 SLC genes in their genomic toolbox (69, 73). It should be noted that VpreB is strongly expressed within pig yolk sacs during fetal gestation, but while it does not appear to function as a surrogate during B cell development, its role is unknown (52, 74). Little is known about the developmental progression of B cells in most vertebrate species, particularly those (*e.g.*, teleost and cartilaginous fish) that utilize a B and T cell-based immune system but lack any obvious surrogate genes for testing their B and T cell receptors during development. Since the order of HC : LC rearrangements likely evolved independently in different species (69), this alternate model of receptor development in pigs provides valuable insight into potential strategies employed by these early vertebrates.

### Newly revealed genes and resolution of erroneous annotations

Creating databases of SLC gene sequences required that we locate (or validate the absence of) SLC genes in genomes from over 60 species. For many of these species, this necessitated a meticulous process of downloading and examining numerous misannotated genes, pseudogenes, or large unannotated regions of a genome. During this process, we found incorrect annotations for two of 63 VpreB1 genes (annotated as Ig Omega or IGLL1) and three of 16 VpreB2 genes (IGLL1 or IGLL5), while 25 of 40 genomes incorrectly annotated IGLL1 as IGLL3, IGLL5, IGLL7, or VpreB1. The majority of genomes annotated VpreB1 and VpreB2 genes using the gene card aliases Ig Iota and Ig Omega, respectively. However, we also identified new locations for two VpreB1 genes (goat and wild yak), three VpreB2 genes (wild yak, horse, and cow), and four IGLL1 genes (manatee, white rhino, camel, and baboon). While painstaking, we located (or confirmed the absence of) VpreB and IGLL1 sequences from nearly all species within our databases using the most current genome assemblies. **Supplementary Table 1** provides the genomic location (or accession number), current annotation, corrected annotation, and taxonomic information for all species in our dataset.

Curiously, during our genome scrutiny we discovered a second VpreB gene in the caecilian (*Rhinatrema bivittatum*) genome that shared greater similarity with VpreB1 and VpreB2 from mammals than to VpreB3. We found sequences from both turtle and alligator (*Alligator mississippiensis*) that similarly aligned to VpreB2 and VpreB1, and the gene annotated as VpreB3 in snake aligned to VpreB2 and VpreB1 as well (see **Supplementary Figures 5**, **6**). While these may simply represent assembly errors within these genomes, evidence of a VpreB gene like those incorporated by SLC in mammals could indicate that the birth of at least one SLC component gene arose earlier and should be examined further.

The dichotomy between VpreB1 and VpreB2 differs in lagomorphs and rodents, where the gene sequences of VpreB2 were nearly identical to those of VpreB1. This homology extends a few hundred base pairs upstream and downstream of the coding regions (75). Both genes are co-expressed during development in mice, though VpreB1 is expressed at higher levels than VpreB2 (8, 75). Dul et al. (75) suggested these two VpreB genes are either under strong selective pressure to conserve their coding regions or one VpreB gene resulted from a more recent duplication of VpreB1 within a short segment of DNA. If the latter is true, these “VpreB2” genes are unrelated to *bona fide* VpreB2 genes that evolved earlier in other groups, and the nomenclature should reflect this more recent evolution (*e.g.*, VpreB1-1, VpreB1-2). Our phylogenetic analysis supports this lack of relatedness, since all lagomorph and rodent VpreB2 sequences branched with VpreB1 sequences from other species (and not the other way around; see **Supplementary Figures 2B**, **6**, **7**). We suggest the VpreB genes in glires be renamed VpreB1-1 and VpreB1-2 to discriminate it from the distinct VpreB1 and VpreB2 genes found in other eutherian mammals.

Finally, recent reports suggest zebrafish genomes contain putative orthologs of both VpreB1 and IGLL1 (76, 77), and single-cell analysis revealed *sid1* expression during early B cell development, suggesting a pre-B cell phenotype in zebrafish (76). We confirmed that both orthologues (*sid1* and *si:ch211-1a19.2*, respectively) contain Ig superfamily domains. Interestingly, these genes are adjacent to each other and occur in the same transcriptional orientation in the zebrafish genome as VpreB1 and IGLL1 are found in many eutherian mammals (see **Figure 3**). However, gene synteny is otherwise not conserved, with no conservation between flanking genes between zebrafish and any tetrapod vertebrate. While sequence similarity between *sid1* and VpreB is low (< 40%), *sid1* most resembles VpreB3 (not VpreB1 or VpreB2) in amino acid sequence conservation and sequence length. Further, a BLAST protein search using the zebrafish *sid1* gene as query against Eutheria (taxid: 9347) returned hits to primarily T cell receptor chains, and unrooted phylogenetic trees place *sid1* firmly within the VpreB3 branch (**Supplementary Figure 6**). Thus, it is more plausible that the zebrafish *sid1* gene is a VpreB3 ortholog. The putative ortholog of IGLL1 (*si:ch211-1a19.2*) shared less than 35% sequence similarity to IGLL1 of eutherians and did not contain the conserved GPRC motif observed within the tail region of IGLL1. A BLAST protein search against Eutheria using the zebrafish *si:ch211-1a19.2* gene as query returned Ig HC or LC hits, though none to IGLL1 sequences. These results suggest that zebrafish do not have a pre-B cell phenotype. Further, zebrafish B cells develop without a distinct pre-B cell stage, and larvae weakly co-express both Igµ and Igκ but not Igµ alone (78). Thus, while mouse B cells undergo two distinct stages of RAG-mediated locus rearrangement (HC followed by LC) that mandates use of a surrogate chain in the pre-B cell stage, zebrafish would not require a surrogate to create functional B cell receptors. Nonetheless, since VpreB3 associates with nascent Igµ during B cell development, the expression of *sid1* during early developmental stages may indicate that *sid1* functions in a similar way as VpreB3 during early B cell receptor development. This suggests the “chaperone” function of VpreB3 likely developed earlier in B cell evolution and may offer an additional explanation for how organisms without SLC make functional receptors during development.

### Relationship between HC and SLC in species with long CDR H3

Pre-BCRs are expected to play a major role in B cell repertoire development of most placental mammals by interacting with the CDR H3 regions of rearranged HC. Crystal structures of human SLC paired with HC demonstrate that C-UR of VpreB forms a flexible probe that covers the CDR H3, thereby blocking antigen-binding sites. Further, residues within UR tail regions of SLC create a CDR H3 sensing site that controls sequence selection within the CDR H3, helping shape the resulting HC repertoire (9, 22). If contact of VpreB/IGLL1 UR tails with CDR H3 helps educate BCR repertoires of eutherian mammals, SLC ultimately may require that CDR H3 be within reach of the probe-like VpreB C-UR. While the structural flexibility of these tails may be able to adapt to CDR H3 of different content and loop lengths (9), cattle present a unique challenge for appreciating SLC/CDR H3 interactions because they assemble a subset of antibodies with an ultralong CDR H3. These CDR H3 can include up to 70 residues that structurally form a unique “stalk and knob” domain that protrudes far from the distal end of the antibody, with antigen binding occurring at the knob (23, 30, 79). For comparison in humans, most CDR H3 are typically 8-16 amino acids in length, and the longest CDR H3 recorded contained 35 residues (30). Nearly all UL antibodies result from recombination of the same V, D, and J gene segments (IGHV1-7, IGHD8-2, and IGHJ2-4) (30, 80, 81). The exceptional length of UL CDR H3 results from a remarkably long germline DH segment, and diversity is driven primarily through somatic hypermutation within the DH-encoded knob domain (23, 30, 80). Thus, it is possible that unusual or specialized CDR H3 regions, like bovine ultralong CDR H3s, may have required co-evolution of specialized SLCs.

We predicted that because the CDR H3 (particularly DH gene segments) of UL antibodies are much longer than usual, the requisite length of VpreB C-UR tails also may have evolved increased length to enable interaction with CDR H3 during testing on the cell surface. Alternatively, we postulated that the altered structure of UL Ab (with the stalk and knob domains protruding from the antibody) prohibits the use of VpreB C-UR as a CDR H3 sensing or sequence selection tool. In this case, VpreB (as part of the pre-BCR) would serve simply to transport HC to the cell surface for assembly with Igα/Igβ signaling molecules during pre-BCR testing. Thus, we examined VpreB1 (n=11) and VpreB2 (n=6) C-UR tail lengths in relation to average and maximum CDR H3 length, particularly the DH segment encoding the longest part of the CDR H3. We found no relationship between average or maximum lengths of VpreB tails and lengths of CDR H3 for either VpreB1 or VpreB2. However, VpreB2, but not VpreB1, tail length strongly correlated with maximum (r = 0.81; p = 0.0499; n=6) DH length, though this relationship disappeared when we removed cattle from the dataset, suggesting the relationship between VpreB2 tail and DH length was biased by the extreme length of cattle DH regions (**Figure 6**). In cattle, CDR H3 (or particular DH portions) are substantially longer than those of other species, and the VpreB1 tail (35 residues) was not remarkably different in length from that of VpreB2 tail (33 residues). Thus, while UL HC may employ VpreB2 for testing at the cell surface, it is possible that the knob region at the tip of CDR H3 cannot be readily bound by either VpreB. Instead, negatively charged VpreB tails may bind conserved regions within the UL antibody structure, for example the positively charged lysine residues in the stalk region, and function in stabilizing the structure during transport to the cell surface rather than helping to shape the UL antibody repertoire. Cattle UL HC, as well as the growing set of vertebrate antigen receptors with extended “reach,” may well require distinct developmental checkpoints and tools than those used by canonical receptors (82).

VpreB (particularly VpreB1) and IGLL1 tail regions contain numerous charged amino acids (**Supplementary Figures 1**-**3**), leading us to examine whether tail length affects the isoelectric point (pI) of VpreB1, VpreB2, or IGLL1 sequences. Indeed, we found that as pI of VpreB1 decreased, its tail length increased (r = -0.31, p = 0.021, n = 56; **Figure 7A**), though the tail region itself did not contribute significantly to this relationship (r = -0.22, p = 0.09, n = 56; **Figure 7B**). We observed these same trends with VpreB2 (VpreB2 pI: r = -0.59, p = 0.026, n = 24; VpreB2 tail pI: r = -0.25, p = 0.24, n = 24; **Figures 7C, D**). Unexpectedly, the pI of IGLL1 tails increased significantly with maximum CDR H3 (r = 0.73, p = 001, n = 11), and this relationship remained even with cow removed from the dataset (r = 0.78, p = 0072, n = 10; **Figure 8**). Because the N-UR tail of IGLL1 forms the VpreB g-strand and one side of the CDR H3 sensing site, perhaps pre-BCR composed of HC with longer CDR H3 require IGLL1 tails to contact alternate residues for proper folding of the protein. Further structural and functional studies from cattle and other non-model species are critical for elucidating these relationships.

### Summary and broader implications

Genomes of all tetrapod groups contained VpreB3 genes, but only eutherian mammal genomes contained genes comprising SLC (VpreB1, VpreB2, and IGLL1). While VpreB1 and IGLL1 genes occurred within nearly all eutherian mammals, we identified VpreB2 genes in only a small subset of species. Additionally, we found evidence of a second VpreB gene in non-eutherian tetrapods, and phylogenetic trees indicate this second VpreB was more closely related to VpreB1/B2 than to VpreB3. This evidence suggests that VpreB2 may have evolved in early tetrapods and subsequently was lost in major mammal radiations, including monotremes, marsupials, and perhaps several eutherian groups. We found that species with longer germline diversifying segments (DH) also have longer VpreB2 tail regions, though it remains unclear whether these longer tail regions are sufficient to function as a CDR H3 sensing tool for bovine ultralong HC antibodies. The use of VpreB simply to aid transport of these unwieldy HC proteins should be explored further. Finally, it is critical to explore alternate strategies for testing receptor functionality (like that seen in pigs), since these alternate strategies may shed light on possible approaches to receptor development in non-tetrapod organisms that lack SLC completely, and these alternate strategies can facilitate an understanding of the constraints limiting repertoire development within vertebrates as a whole.

The results reported in this study emphasize the value of evolutionary comparative analyses to assess immunogenetic and functional studies of the immune system. While research in mouse and human immune systems may directly benefit human health and therapeutics, studies in evolutionarily distant species often affords essential and unexpected context for the molecules and mechanisms involved in disease (83). By examining gene synteny and phylogeny within eutherian mammals, we evaluated whether the (projected) function of SLC components changes based on the length of an immunoglobulin CDR H3. We specifically assessed species like cattle that manufacture ultralong CDR H3 antibodies that are unlikely to be trained by a canonical SLC during VDJ rearrangement. Previously, we reported that nurse shark T cells employ AID-mediated somatic hypermutation (SHM) to diversify the primary αβ T cell repertoire during thymic development. Since SHM is a mechanism typically used only by activated B cells to affinity mature antigen receptors, the use of SHM by T cells indicated that ancestral lymphocytes did not have a clear division of B and T cell repertoire diversification mechanisms (63, 64). Our multiple dimension immunogenetic analyses mirror the “5-dimensional” approach taken by Ghorbani et al. (83) to review proteins within the AID/APOBEC family across deuterostome animals. The authors integrated 3-dimensional structures with knowledge of real-time conformational and evolutionary time shifts (4^th^ dimension) and biological function (5^th^ dimension) and suggest that, since the greatest evolutionary diversity of all proteins lies in earlier-evolved organisms, studies of these organisms can uncover fundamental biological insights in structural biology, immunology, and cancer research (23). Thus, evolutionary comparative studies are critical for providing the context for understanding basic immunological processes.

## Data availability statement

The datasets presented in this study can be found in online repositories. The names of the repository/repositories and accession number(s) can be found in the article/**Supplementary Material**.

## Author contributions

JKH, MFC, and VVS contributed to conception and design of the study. CC, ARK, JKH, and JAO organized the database. JKH and JAO performed the statistical analysis. JAO and JKH wrote the first draft of the manuscript. All authors contributed to manuscript revision, read, and approved the submitted version.

## Funding

Work was supported by grants NIH R01 HD088400 and NIH R01 GM105826 to VVS and NSF IOS 165870 to MFC.

## Acknowledgments

We thank the Smider and Criscitiello labs for their helpful discussions.

## Conflict of interest

The authors declare that the research was conducted in the absence of any commercial or financial relationships that could be construed as a potential conflict of interest.

## Publisher’s note

All claims expressed in this article are solely those of the authors and do not necessarily represent those of their affiliated organizations, or those of the publisher, the editors and the reviewers. Any product that may be evaluated in this article, or claim that may be made by its manufacturer, is not guaranteed or endorsed by the publisher.

## Abbreviations

SLC, Surrogate light chain; Ig, Immunoglobulin; IgL, Lambda light chain locus; HC, Heavy chain; LC, Light chain; BCR, B cell receptor; TCR, T cell receptor; V, Variable gene segment; DH, Diversity gene segment of the heavy chain; J, Joining gene segment; C, Constant region; CDR H3, Third complementarity-determining region of the heavy chain; C-UR, Carboxy-terminal unique region; N-UR, Amino-terminal unique region; pI, Isoelectric point; IGLL1, Immunoglobulin lambda-like polypeptide 1 gene, which encodes the λ5 protein; PTCRA, Pre-TCRα; RAG, Recombination activating genes; UL, Ultralong.
