# Supplementary material for: Evolution of surrogate light chain in tetrapods and the relationship between lengths of CDR H3 and VpreB tails
Source: Front Immunol. 2022 Oct 13;13:1001134. doi: 10.3389/fimmu.2022.1001134 (PMC9614664; doi:10.3389/fimmu.2022.1001134)

Supplementary Material

Supplemental Figure 1. VpreB1 genes are conserved within eutherian mammals. Amino acid alignment of VpreB1 sequences from 55 species of mammals (species names are colored to match their placement on the tetrapod cladogram in Figure 2). Residues located within the variable region of the gene are shaded based on similarity using a Blosum62 scoring matrix (threshold = 1, gaps ignored; highlights indicate similarity: black = 100% similar; dark gray = 80 – 100% similar; light gray = 60 – 80% similar; white = <60% similar). Charged residues within the tail region are highlighted blue (basic: arginine (R), pI=10.8; lysine (K), pI=9.8; histidine (H), pI=7.6) or red (acidic: glutamic acid (E), pI=3.2; aspartic acid (D), pI=3.0). We removed signal peptides from alignments. Percent sequence similarity to the aardvark (left, read top to bottom) or human (right, read bottom to top) VpreB1 sequence is shown to the right of the alignment (green: variable region; blue: N-UR tail).

Supplemental Figure 2. VpreB2 is the least conserved surrogate light chain gene within eutherian (placental) mammals. [A] Amino acid alignment of VpreB2 sequences from 23 species of mammals (species names are colored to match their placement on the tetrapod cladogram in Figure 2). Rabbit and rodent species below the break are more related to VpreB1 than VpreB2 in other species. Percent sequence similarity to the aardvark (left, read top to bottom) or human (right, read bottom to top) VpreB2 sequence is shown to the right of the alignment. **[B]** Comparison of VpreB1 (top) and VpreB2 (bottom) sequences from rabbits and rodents are highly related. Percent sequence similarity of VpreB1 and VpreB2 for an individual species is shown to the right of the alignment. Residues located within the variable regions are shaded based on similarity using a Blosum62 scoring matrix (threshold = 1, gaps ignored; highlights indicate similarity: black = 100% similar; dark gray = 80 – 100% similar; light gray = 60 – 80% similar; white = <60% similar). Charged residues within the tail regions are highlighted blue (basic: arginine (R), pI=10.8; lysine (K), pI=9.8; histidine (H), pI=7.6) or red (acidic: glutamic acid (E), pI=3.2; aspartic acid (D), pI=3.0). We removed signal peptides from alignments (green: variable region; blue: N-UR tail).

Supplemental Figure 3. IGLL1 genes, which encode λ5 protein, are conserved within eutherian mammals. Amino acid alignment of λ5 sequences from 39 species of mammals (species names are colored to match their placement on the tetrapod cladogram in Figure 2). Charged residues within the tail region are highlighted blue (basic: arginine (R), pI=10.8; lysine (K), pI=9.8; histidine (H), pI=7.6) or red (acidic: glutamic acid (E), pI=3.2; aspartic acid (D), pI=3.0). Residues located within the constant region of the gene are shaded based on similarity using a Blosum62 scoring matrix (threshold = 1, gaps ignored; highlights indicate similarity: black = 100% similar; dark gray = 80 – 100% similar; light gray = 60 – 80% similar; white = <60% similar). We removed signal peptides from alignments. Percent sequence similarity to the manatee (left, read top to bottom) or human (right, read bottom to top) Lambda5 sequence is shown to the right of the alignment. Sequence regions are indicated by the colored areas above the consensus sequence (blue: C-UR tail (top); yellow: variable region (bottom))

Supplemental Figure 4. VpreB3 genes are conserved across tetrapod species, with 63% of residues common between caecilian (amphibian) and human (mammal) sequences. Amino acid alignment of VpreB3 sequences from 64 species of tetrapods (species names are colored to

match their placement on the tetrapod cladogram in Figure 2). Residues located within the variable region of the gene are shaded based on similarity using a Blosum62 scoring matrix (threshold = 1, gaps ignored; highlights indicate similarity: black = 100% similar; dark gray = 80 – 100% similar; light gray = 60 – 80% similar; white = <60% similar). Charged residues within the tail region are highlighted blue (basic: arginine (R), pI=10.8; lysine (K), pI=9.8; histidine (H), pI=7.6) or red (acidic: glutamic acid (E), pI=3.2; aspartic acid (D), pI=3.0). We removed signal peptides from alignments. Percent sequence similarity to the caecilian VpreB3 sequence (left, read top to bottom) or human VpreB3 sequence (right, read bottom to top) is shown to the right of the alignment (green: variable region; blue: N-UR tail).

Supplemental Figure 5. Immunoglobulin (Ig) domains of PTCRA genes are highly conserved among eutherian mammals (sequences share at least 83% of residues), while cytoplasmic tail (Ct) regions are highly divergent (sequences share roughly 20% of residues and differ considerably in length). Amino acid alignment of PTCRA sequences from 63 species of tetrapods (species names are colored to match their placement on the tetrapod cladogram in Figure 2). Residues are shaded based on similarity using a Blosum62 scoring matrix [threshold = 1, gaps ignored; highlights indicate similarity (black = 100% similar; dark gray = 80 – 100% similar; light gray = 60 – 80% similar; white = <60% similar)]. Percent sequence similarity to the turtle (left, read top to bottom) or human (right, read bottom to top) PTCRA sequence is shown to the right of the alignment. Sequence regions are indicated by the colored areas above the consensus sequence [top: blue: immunoglobulin (Ig) domain; gold: connecting peptide (Cp); green: transmembrane (Tm) domain; bottom: gray: cytoplasmic tail (Ct)].

Supplemental Figure 6. Phylogenetic analyses of VpreB1, VpreB2, and VpreB3 sequences indicate that VpreB3 originated first in the genome, with VpreB2 and VpreB1 diverging more recently. Phylogenetic relationships were determined between all VpreB sequences using the Geneious tree builder (Geneious Prime v. 2022.0.1, Biomatters Ltd) with default settings and a bootstrap resampling method of 2000 replicates. Branch colors were coordinated with those representing each VpreB gene shown in Figure 2 (pink: VpreB3; orange: VpreB-like (non-mammalian tetrapods); green: VpreB2; blue: VpreB1). Branch labels represent percent consensus support using majority greedy clustering. Note that VpreB3 from snake (shown in orange) also groups with VpreB2, indicating greater similarity to the VpreB-like sequences we found in non-mammalian tetrapods rather than to VpreB3.

Supplemental Figure 7. Rodent VpreB2 genes are more similar to VpreB1 (> 94%) than to VpreB2 from other species (<60%), suggesting that rodent VpreB2 stems from a more recent duplication event and may not exhibit the same function as VpreB2 of other Eutherian mammals. Phylogenetic relationships were determined between VpreB1 and VpreB2 sequences using the Geneious tree builder (Geneious Prime v. 2022.0.1, Biomatters Ltd) using default settings with a bootstrap resampling method of 1500 replicates. Branch colors were coordinated with those representing each SLC gene shown in Figure 2 (orange: VpreB2-like (non-mammalian tetrapods); green: VpreB2; blue: VpreB1). Branch labels represent percent consensus support using majority greedy clustering.

Supplemental Figure 8. A second VpreB gene found in caecilian, turtle, and alligator genomes share greater similarity with VpreB1 and VpreB2 genes (especially

Cetartiodactylan VpreB2) than with VpreB3, suggesting VpreB2 may have evolved earlier in the tetrapod radiation. Amino acid alignments of a VpreB2-like gene sequences **[A]** found in three non-amniote tetrapods; **[B]** compared to VpreB3 gene sequences from five non-amniote tetrapods; and **[C]** compared to VpreB2 gene sequences from 23 eutherian mammals (species names are colored to match their placement on the tetrapod cladogram in Figure 2). Residues located within the variable region of the gene are shaded based on similarity using a Blosum62 scoring matrix (threshold = 1, gaps ignored; highlights indicate similarity: black = 100% similar; dark gray = 80 – 100% similar; light gray = 60 – 80% similar; white = <60% similar). Charged residues within the tail region are highlighted blue (basic: arginine (R), pI=10.8; lysine (K), pI=9.8; histidine (H), pI=7.6) or red (acidic: glutamic acid (E), pI=3.2; aspartic acid (D), pI=3.0). We removed signal peptides from alignments. Percent sequence similarity to the first species (left, read top to bottom) or the last species (right, read bottom to top) is shown to the right of the alignment (green: variable region; blue: N-UR tail).

Supplemental Figure 9. **Locus synteny of VpreB3 in toad and caecilian, showing the absence of VpreB3 in coelacanth and skate**. VpreB3 is shown in pink and the second VpreB gene found in caecilian is colored yellow. Polygons contain numbers representing gene names (see Supplemental Table 2B for gene names represented by each numbered polygon). A solid line represents contiguous genomic sequence (chromosome or unplaced scaffold) and is denoted by the chromosome (Chr) or scaffold (Scf) to which it belongs. A line ending in a diamond shape indicates the end of a chromosome or scaffold and no further genes occur in that direction. Orientation of chromosomes or scaffolds is indicated by the 5' or 3' labels at the ends of the solid lines, and gene polygons point in their transcriptional direction. Colored lines above the polygons denote syntenic gene blocks, and lines containing an arrow indicate the syntenic block is inverted compared to toad (*Bufo bufo*). The location of the lambda light chain locus (present only in thorny skate) is indicated by a gray rectangle. Orthologs between species are aligned vertically, but distances are not to scale.

Supplemental Figure 10. Locus synteny of pre-T cell receptor alpha (PTCRA) chain genes in tetrapod vertebrates. PTCRA is shown in blue. Syntenic genes found in two or more species are colored light gray. A solid line represents contiguous genomic sequence (chromosome or unplaced scaffold) and is denoted by the chromosome (Chr) or scaffold (Scf) to which it belongs. Orientation of chromosomes or scaffolds is indicated by the 5' or 3' labels at the ends of the solid lines, and gene polygons point in their transcriptional direction. Orthologs between species are aligned vertically, but distances are not to scale. (RPL7L1: Ribosomal Protein L7 Like 1; CNPY3: Canopy FGF Signaling Regulator 3; POLR1B: Polymerase (RNA) I Subunit B; EPHB2: Ephrin Type-B Receptor 2; ATL2: Atlastin GTPase 2)

Suppl. Figure 1

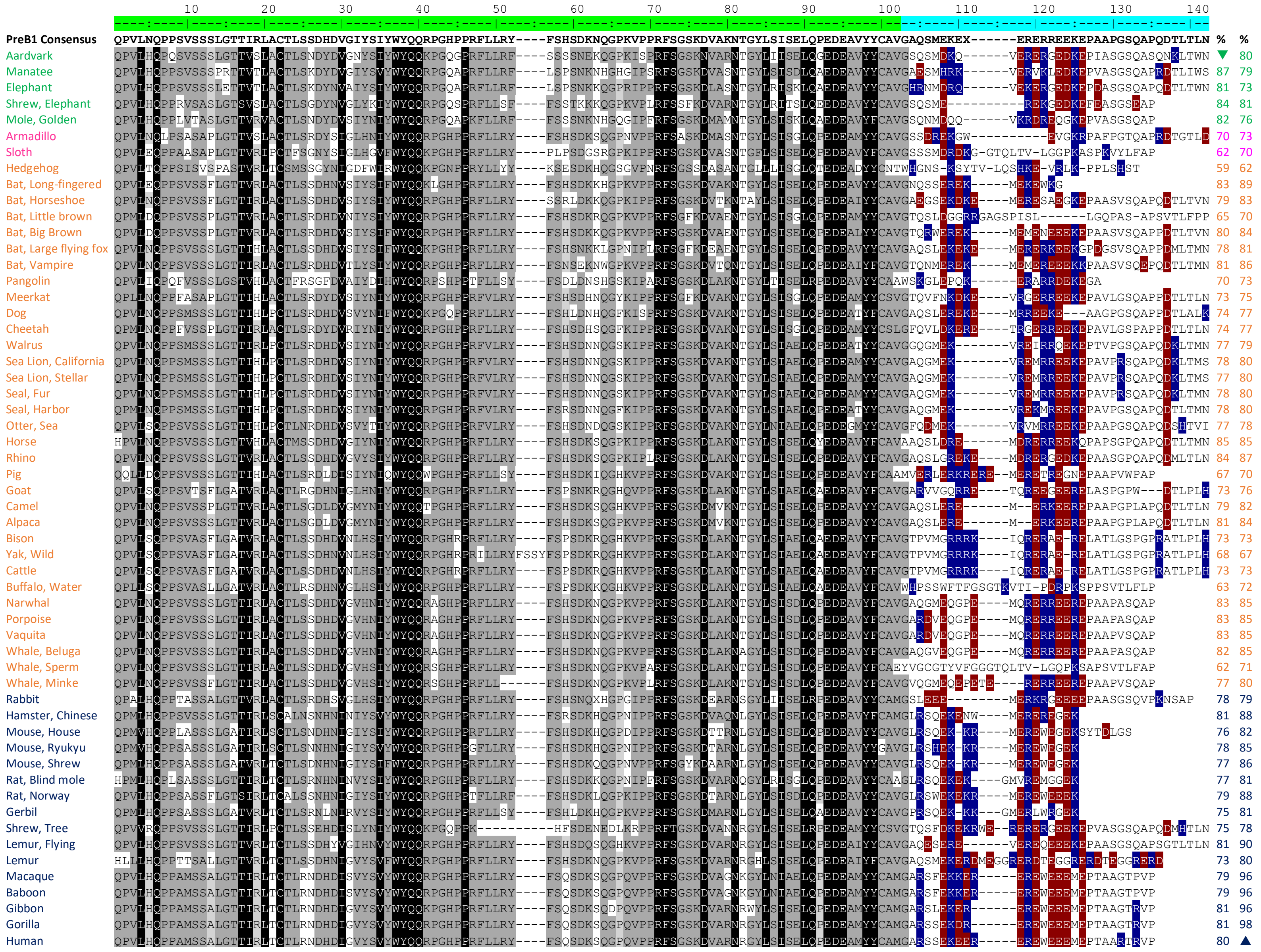

Suppl. Figure 2

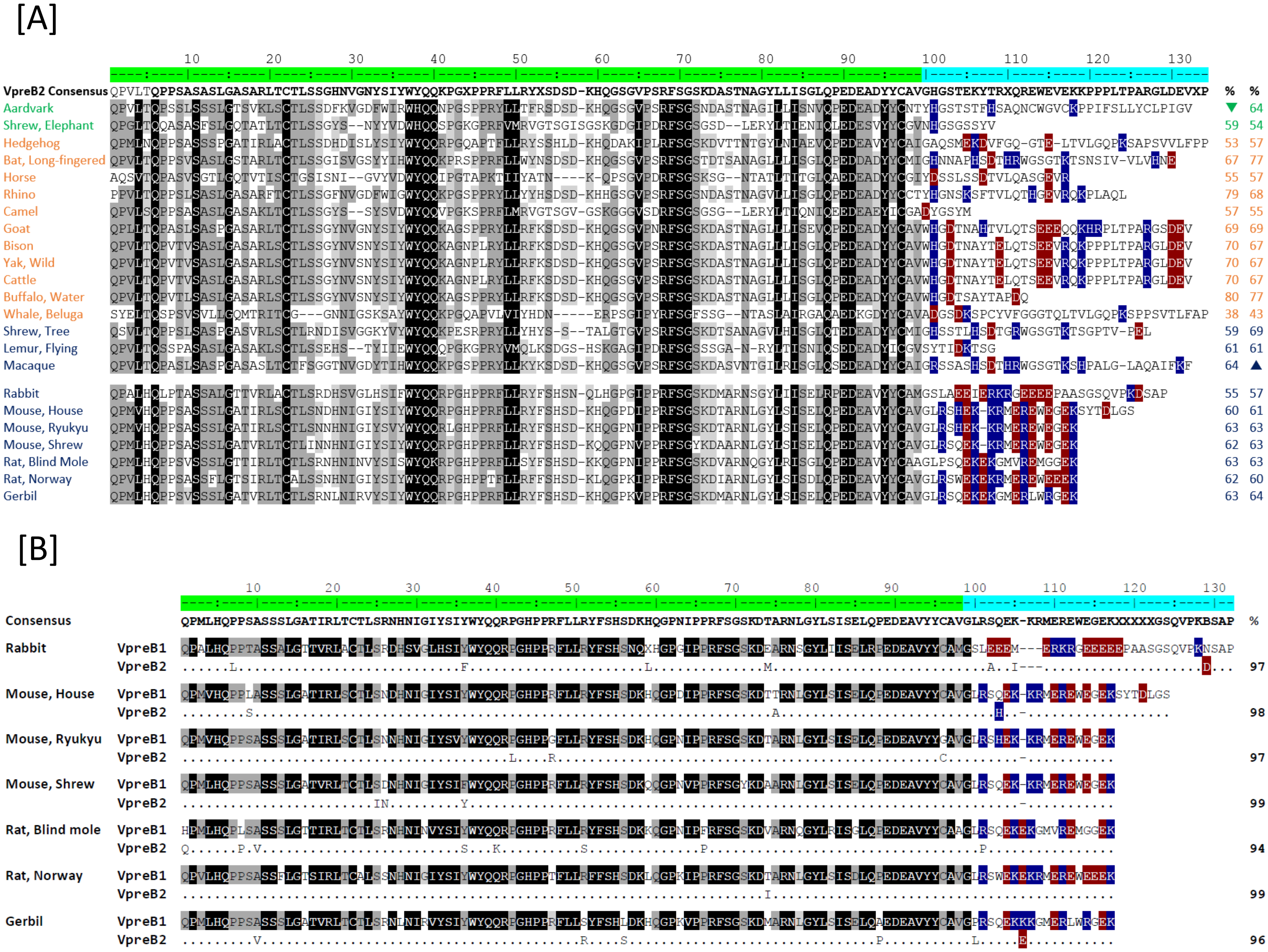

Suppl. Figure 3

```
                           10        20        30        40        50        60        70        80        90       100
                  ----:----|----:----|----:----|----:----|----:----|----:----|----:----|----:----|----:----|----:----|
IGLL1 Consensus   LLGLAVGAHGLLPPT-AAPQSRAPGPGAPAGS-----SPWSLWG--RFLLQPGPRGAGPRCWPGGFWSEPQSPWYVFGSGTQLTV--LGQPKSAPSVTLF
Manatee           LLLLALSPLGLWPLT-ATPRSSAPGPRAPAGGSRS--RLRSLWG--RFLLWPGPQGTGPRCWPHQFWYEPESPWYVFGSGTQLTV--LGQPKASPSVTLF
Mole, Golden      ILLLGLGALGLRRPT-ATRRSSAPRPGAPEVDSRS--HLRSLRG--RFSLWPGPQSAGPRCWPGQFWAESPSSWFIFGSGTHLYV--LGQPKTSPMVTLF
Armadillo         LLALVMGAHSVLPPT-AAPPSTPGPRGTAGSSRSSAGNLQSLWD--RSPLKPTPQGEGPRCWLRGFLSESQAPWYVFGSGTQLTV--LGQPKASPIIHLF
Bat, Big brown    LLGLAVGTHGLLSST-TAPRSRAPSAGAPTRS-----SPRSLWD-SRFPFRTGPQGTGPRCWPGAFWSDLQSPWYVFSSGTQLIV--LGQPTSAPSVTLF
Bat, Flying fox   LLGLAVGTHGLSLPT-AAPRGRAPGPGAPTRS-----SPWSLWD--RSLLRTGPQEAGPRCWPGGLWSNLQSPWYVFSSGTQLIV--LGQPKSAPLIILF
Meerkat           LLGLAVGAHGQLPPTTAAPPSRAPGPGAPAGS-----SPRSLWD--RFLFWPGPHGAGPRCWPDGFWSETRSPWFVFSEGTQLTV--LGQPKSAPSVTLF
Dog               LLGLAVGAHGRLPPTTAAPPGSAPGPRAPGGS-----SSRSLWD--RFLFWPGPHGAGPRCWPGGFWPETQSPWFVFSEGIELTV--LGQPKASPSVTLF
Sea lion, Cal     LLGLAVGAHGRLPPVTVAPPNSTPDPGAPAGS-----SSRSLWD--RFLFWPGPRGAGPRCWPGGFWPETQSPWFVFSEGTQLTV--LGQPKSAPLVTLF
Sea lion, Stellar LLGLAVGAHGRLPPVTVAPPNSTPDPGAPAGS-----SSRSLWD--RFLFWPGPRGAGPRCWPGGFWPETQSPWFVFSEGTQLTV--LGQPKSAPLVTLF
Seal, Harbor      LLGLAVGAHGRLPPLPVDPPNSTPDPGAPAGS-----SSRSLWD--RFLFWPGPQGAGPRCWPGGFWPETQSSWFVFSEGTQLTV--LDQPKSAPLVMLF
Otter, Sea        LLGLAVGAQGRLPPTTVAPPNSTPEPRAPAGS-----SSRSLWD--RFLFWPGPHGAGPRCWPGGFWPETQSPWYVFSDGTQLIV--LGQPKSTPSVTLF
Horse             LLGLAVGTHGLLPPT-AAPRGKTPGPGAPAGS-----SPWRLWD--RFPLRPGPRGTGPRCWPGGFWSDVQPPWYIFSEGTQLTV--TGGPKSAPSVTLF
Rhino             LLGLAVGAHGLLPPT-AAPRGRTPGPGAPGRS-----SPWSVWD--RS-LRPGPRGAGPRCWPGGFWSETQSPWYVFSEGTQLTVLDTGQPKSAPSVSLF
Pig               LLGLAMGAHGLLPPT-TAPQSRAPGTEAPARS-----SPWRLWD--RFLLRPPPRGTGPRCWPARFWSEPQPPWVVFGGGTRLTV--LGQPKAAPTVTLF
Goat              LLGLAVGAHGLLLPT-AAPQSTAPRMEAPVSI-----TPS------RFPLRPDPRGTGPRCWPG-VWSEASLPWYVFGSGTEVTI--SGKPKSAPSVTLF
Camel             LLGLAVGAHGLLPST-AAPPNRAPRTEASAES-----SPWSRWD--RSPLQPGPRGAGPRCWPGRFWSEPQSLWYVFGSGTHLTV--LGQPKAAPSVTLF
Bison             LLGLVVGAHGLLPPT-AAPQSTAPRMEAPVSI-----TPS------RFPLRPGPRGPGPRCWRG-VWPEGPSAWFTFGSGTKVTI--PDQPKFPPSVTLF
Yak, Wild         LLGLAVGAHGLL----AAPQSPAPRMEAPVSI-----TPS------RFPLRPGPRGPGPRCWRG-VWSEGPSAWFTFGGGTKVTI--PGQPKSPPSVTLF
Buffalo, Water    LLGLAVGAHGLLPPT-AVPQSTAPRMEAPSGK-----SPWSLRG--RLPLRPSPRGTGPRCWPG-VWSGAPSSWFTFGSGTKVTI--PDRPKSPPSVTLF
Cattle            LLGLAVGAHGLLPPT-AAPQSTAPRMEAPSGK-----SPWSLRG--RFPLRPGPRGPGPRCWRG-VWPEGPSAWFTFGSGTKVTI--PDQPKFPPSVTLF
Porpoise          VLGLAVGAHGLLPPT-PAPQSTAPRKETPAGS-----SPWSLWH--SSLLHPGPRGTGPRCWPGRFWSESQLPWYVFGGGTQLTV--LGQPKSAPSVTLF
Vaquita           VLGLAVGAHGLLPPT-PAPQSTALRKETPAGS-----SPWSLWH--SSLLHPGPRGTGPRCWPGRFWSESQLPWYVFGGGTQLTV--LGQPKSAPSVTLF
Whale, Beluga     VLGLAVGAHGLLPPT-PAPQSTAPRKETPAGS-----SPWSLWH--SSLLHPGPRVTGPRCWPGGFWSESRSPWYVFGGGTQLTV--LGQPKSAPSVTLF
Whale, Sperm      LLGLAVGAHGLLPLT-PAPQSTAPRKETPAGS-----SPWSLWH--RSPLRPGPRGTGPRCWPDGFWSESQSLRYVFGGGTQLTV--LGQPKSAPSVTLF
Rabbit            LLGLVVGAQDLWTPT-AAPQSRALGPGGPVGSSRF--NLRIPWH--RLPLQPGPQEAGPRCWPHGFWSESQSRWYIFGSGTQLSV--TGQPVVAPLVTLF
Hamster, Chinese  LLGLVDGVQH-------ARRSRAMGPGASVGSSRS--SLWTLPG--RFLFRAIPRETGPRCWPHGFLSKPQ-SWYIFGSGTQVTV--LGQPKSDPLVTLF
Mouse, House      LLGLVDGVHHILSPS-SAERSRAVGPGASVGSNRP--SLWALPG--RLLFQIIPRGAGPRCSPHRLPSKPQ-FWYVFGGGTQLTI--LGQPKSDPLVTLF
Mouse, Ryukyu     LLGLVDGVHHIFSPS-SAERSRAVGPGASVGSSRP--SLWALPG--RFLLQIIPRGAGPRCSPHRLPSKPQ-FWYVFGGGTQLTI--LGQPKSDPLVTLF
Mouse, Shrew      LLGPVDGVHHILSPS-SAQRSGAVGPGASVGSSRP--SLWALPG--RFLFQIIPRGAGSRCLPPRRPSESQ-YWYVFGGGTQLTI--LGQPKSDPLVTLL
Rat, Blind mole   MLGLVEGVQGLLSPS-SARGSRPLDRGALVGRSRS--SLWILPG--RFEFQLIPQGTCSRCWPRGSPSEPKSSWYVFGSGTQLTV--LGQPKAAPLVTLF
Rat, Norway       LLGLVDGVHHILSPS-SAQRGRAVGPGASVGSSRS--SLWTLPG--RFLFQIIPRGAGPRCWPHRLPSKSQ-LWYVFGSGTQLTI--LGQPKSDPLVTLF
Gerbil            LLGLVDGVQHVLSPS-SAQRSRALGSGSSGGSSRP--RLWALPG--RFLFQIIPWRTGPRRWPYGRPSEPQ-PCLSFGDGTRLTI--LGQPKSDPLVTLF
Shrew, Tree       LLGLVVGTYGLLPLT-TAPRREARGPRGWAGS-RS--SPRSQWGSPRFLLRPVPQGAGPRCWPHGFWSE--SPWFVFGSGTQLHV--LGQPKVTPTITLF
Lemur, Flying     PLGLAVGIRGLVSPM-AAAWSRAPGLGTRAGSSWS--SLQRLWG--RILLQPSPWRAGPRCWPCGFWSEPQSPWNVFGGGTQLTV--LGQPKAAPSVTLF
Lemur             LLGLAVGTHGFLSST-EAPRSRAPGPGARAGSSRS--SPRIPWS--RFLLQPSPRGAGARCWPRGFWSEPQSLWYIFGRGTQLTI--LGQPKAAPSVTLF
Baboon            LLGLALVTYGLVHPT-AAPQSRALGPGAPGGSSRS--SLRSRWG--RFLLQHGSW-TGPRCWPRGFQSKHNTLRHVFGSGTQLTV--LGQPKATPSVTLF
Gibbon            LLGLAVVTHGLLRPT-AAPQSRALGPGXPGGSSRS--SLRSRWG--RFLLQRGSW-TGPRCWPRGFQSKHNSVTHVFGSGTQLTV--LTQPKAAPSVTLF
Gorilla           LLGLAVVTHGLLRPT-AAPQSRALGPGAPGGSSRS--SLRSRWG--RFLLQRGSW-TGPRCWPRGFQSKHNSVMHVFGSGTQLTV--LSQPKATPSVTLF
Human             LLGLAVVTHGLLRPT-AASQSRALGPGAPGGSSRS--SLRSRWG--RFLLQRGSW-TGPRCWPRGFQSKHNSVTHVFGSGTQLTV--LSQPKATPSVTLF
```

```
                         110       120       130       140       150       160       170       180       190
                  ----:----|----:----|----:----|----:----|----:----|----:----|----:----|----:----|----:----|----
IGLL1 Consensus   PPSSEELQANKATLVCLISDFYPGSVTVAWKADGTPVTQGVETTQPSKQSNNKYAASSYLXLTPDQWKSHSSYSCQVTHEGSTVEKTVAPAECS     %   %
Manatee           PPSSEELQANKATLVCLMNDFYPDAVTVTWKEDGTTISQGIETTKPSKQSNNKYAASSYLTLKPAQWRSHKSYSCQVTHEGSTVEKKVAPAECA    ▼   78
Mole, Golden      LPSSQELKTNKATLVCLISDFYPGTMTVAWKADGTTITQSVETSKPSKQNNNKYAASSYLTLTAAQWMSHDSYSCLVTHEGKTMEKKVIPAECA    79  71
Armadillo         PPSPKELKTNKATLVCLVSAFYPGSVAVKWKRDGRDYTEGVQTTKPSKQTDNKYAASSYLSLTPAQWRNADRYSCQVTHEGSTVEKSVSPTQCS    71  70
Bat, Big brown    PPSPEELQTNKATLVCLISDFYPGSVTVAWKADGSPVTQGVETTAPSKQSNNKYAASSYLSLTPDKWRSGGSYSCQVTHEGGTVEKAVVPAECS    74  74
Bat, Flying fox   PPSSEELRTNKATLVCLISDFYPSNMTVAWKANGSPVTQGVETTKPLKQSNNKYAASSYLSLTPDKWKSSSSFSCQVTHEGSTVEKTLAPSQCF    74  75
Meerkat           LPFSEELRA-KSTLVCLISDFHPSTLVVTWKVDGTPITQGVETTKPFKQSNNKYLASSYLSLSPDKWKSHRSFTCQVMHEGSTVEKTVVPAECP    76  75
Dog               LPLSGELGADKATLVCLISDFYPSGVTVAWKADGSPVTQGVETTKPSKQSNNKYAASSYLSLTPDKWKSHSSFSCLVTHEGSTVEKKVAPAECL    77  73
Sea lion, Cal     LSFSEELGANKATLVCLISDFYPSSLTVAWKVDGSNVTQGVETTKPFIQSNNKYAASSYLSLSPDKWKSYSNVSCLVTHEGSTVEKKVVPAECS    75  68
Sea lion, Stellar LSFSEELGANKATLVCLISDFYPSSLKVAWKVDGSNVTQGVETTKPFIQSNNKYAASSYLSLSPDKWKSYSSVSCLVTHEGSTMEKKVVPAECS    74  68
Seal, Harbor      LSFSEELGANEATLVCLVSDFYPSGVTVAWKADGITVTQGVETTKPSKQSNNKYAASSYLSLTPAKWKSYSSVSCLVTHEGRTVEKKVVPAECS    74  67
Otter, Sea        RTFS-EITDNKATLVCLISDFYPSSVMVAWKADGSPVTQGVETTKPSKQSNNKYVASSYLSLSPDMWKSHSSFSCLVTHEGKTVEKKVVPSQCS    74  68
Horse             PPSSEELSTNRATVVCLISDFYPSNLTVVWKANGTPITQGVEITKPLKQSNNKYAASSYLTLSPAMWKSYSSISCQVTHEGSTVEKTVASSQCS    75  74
Rhino             PPSNEELSANKATLVCLISDFYPSDLTVAWKADGTAITQGVETTKPSKQSNNKYAASSYLTLTPDKWKSHSSFSCQVTHEGSTVEKTVVPEECS    76  75
Pig               PPSPEELSTNKATLVCLISDFYPGAVTVTWKAGGTTITQGVDTSQPSKQSNNKYAASSYLALSASDWKSSSGFTCQVTHQGNTVEKTVMPSECA    77  73
Goat              PPSTEELSTNMATLVCLISDFYPGNVTVAWKADGTPVTRGVVTSQASKQSNSKYVASSYLTLTGSEWNPKSSYSCEVTHEGSTVTKTVKPSECS    71  70
Camel             PPSSEELKANKATLVCLISDFYPGNVTVAWKQDGTTVTQGVETTKPSKQSNNKYAASSYLTLSPSTWKSHRSYSCRVTHEAGTVEKTVSPSQC     79  75
Bison             PPSTEELSTYTATLVCLISDFYPGNVTVAWKADGSPVTRGVVTSQASQQSSSKYVASSYLTLTGSEWKPKSSYSCEVTHEGSTVTKTVKPSACS    67  66
Yak, Wild         PPSTEELSTNTATLVCLISDFYPGNVTVAWKADGSPVTRGVVSSQASQQSNSKYAASSYLSLTGSEWKPKSSYSCEVTHEGSTVTKTVKPSACS    68  67
Buffalo, Water    LPSTEELSTNMATLVCLISDFYPGSMTVAWKADWHPVTRGVVTSQASKQSNSKYAASSYLTLMGSEWKPKSSYSCEVTHEGSTVTKTVKPSACS    71  67
Cattle            PPSTEELSTYTATLVCLISDFYPGNVTVAWKADGSPVTRGVVTSQASQQSSSKYVASSYLTLTGSEWKPKSSYSCEVTHEGSTVTKTVKPSACS    71  68
Porpoise          APSTEELKANKATLVCLINDFYPGSVTVAWKAGSTTITRGVETAQPSKQSNSKYAASSYLALTASEWESYESVSCQVTHDGRTVEKTVASSGCP    76  70
Vaquita           APSTEELKANKATLVCLINDFYPGSVTVAWKAGSTTITRGVETAQPLKQSNSKYAASSYLALTASEWESYESVSCQVTHDGRTVEKTVASSGCP    75  70
Whale, Beluga     APSTEELKANKATLVCLINDFYPGSVMVAWKAGSTTITSGVETAQPSKQSNSKYAASSYLALTASEWRSYDRVSCQVTHEGSTVEKTVASSGCP    74  70
Whale, Sperm      APSTEELKTNKATLVCLINDFYPGSVTVAWKAGSTAITRGVETTQPLKQSNGKYAASSYLALTASEWKSYDRVSCQVTHEGSTVEKTVASSGCP    74  71
Rabbit            PPSSEELKANKATLVCLISDFYPGTVTVAWKADGATITQGMDFTQPSKQSNNKYMASSFLSLSRGQWTSHSRFICQVTHEGKTMEKSVAPAHCP    73  73
Hamster, Chinese  LPSLYDLQANKATLVCLVSEFYPGTLVVTWKIDGIPVTQGVETTQPSKQTDNKYVTSSYLTLTSHQWTPHSRYSCQVIHEGNTVEKSVSPAECS    70  72
Mouse, House      LPSLKNLQANKATLVCLVSEFYPGTLVVDWKVDGVPVTQGVETTQPSKQTNNKYMVSSYLTLISDQWMPHSRYSCRVTHEGNTVEKSVSPAECS    70  71
Mouse, Ryukyu     LPSLKNLQANKATLVCLVSEFYPGTLVVDWKVDGVPVTQGVETTQPSKQTNNKYMVSSYLTLISDQWMPHSRYSCRVTHEGNTVEKSVSPAECS    71  71
Mouse, Shrew      LTSFKNLQANKATLVCLVSEFYPGTLVVDWKVDGIPVTQGVETTQPAKQTNGKYMTSSYLTLISDQWMPHSRYSCRVTHEGNTVEKSVSPAECA    66  68
Rat, Blind mole   LPSLKEIQANKYTLVCLVSEFYPGALVVAWKVDGIPVTQGVETTQPFKQTNNKYIASSYLTLMSDQWTPHSRYSCQVTHEGTTVEKSMSPAECS    69  70
Rat, Norway       LPSLKNLQVKKATLVCLVSEFYPGTLVVDWKVDGIPVTQGVETTQPSKQTNNKYVASSYLTLMSDQWMPHSRYTCQVTHEGNTVEKSVSPAECS    70  72
Gerbil            LPSLKNFQAYKATLVCLVSEFYPGTLVVDWKVDGIPVTQGVETTQPSKQTNNKYVVSSFLTLISDQWTPHSRYSCQVTHEGNIVEKSVSPAECS    66  69
Shrew, Tree       APSREMLRAGKAILVCLISDFYPGSVTVAWKVNGTPVSQGVETSQPSKQSNGRYAASSLLSLSARQWGPRSAFTCQVTHEGSTVEKSVTPAGCS    74  72
Lemur, Flying     PPSSEELKDNKATLVCLMSDFYPGAVTVAWKADGTTVTQGVETTKPSKQSNNKYGASSYLTLSPQQWNSHKSYSCQVTHEGSTVEKTVAPAECS    78  79
Lemur             PPSSEELQANKATLVCLMSDFYPGAVSVAWKADGSAVTQGVETTQASKQSNGKYAASSYLSLSPAQWKAGGRFSCQVTHEGSTVEKTVAPAECA    79  79
Baboon            LPSSEELQANKATLVCLMSDFYPGILTVTWKADGTPITQGVEMTMPSKQSNNKYAASSYLSLTPEQWRSHRSYSCQVTHEGSIVEKTVTPAECS    79  94
Gibbon            PPSSEELQANKATLVCLMNDFYPGSLRVTWKADGTPITQGVEMTTPSKQSNSKYAASSYLSLTPEQWRSRRSYSCQVTHEGSTVEKTVAPAECS    79  97
Gorilla           PPSSEELQANKATLVCLMNDFYPGILTVTWKADGTPITQGVEMTTPSKQSNNKYAASSYLSLTPEQWRSRRSYSCQVMHEGSTVEKTVAPAECS    79  99
Human             PPSSEELQANKATLVCLMNDFYPGILTVTWKADGTPITQGVEMTTPSKQSNNKYAASSYLSLTPEQWRSRRSYSCQVMHEGSTVEKTVAPAECS    78  ▲
```

Suppl. Figure 4

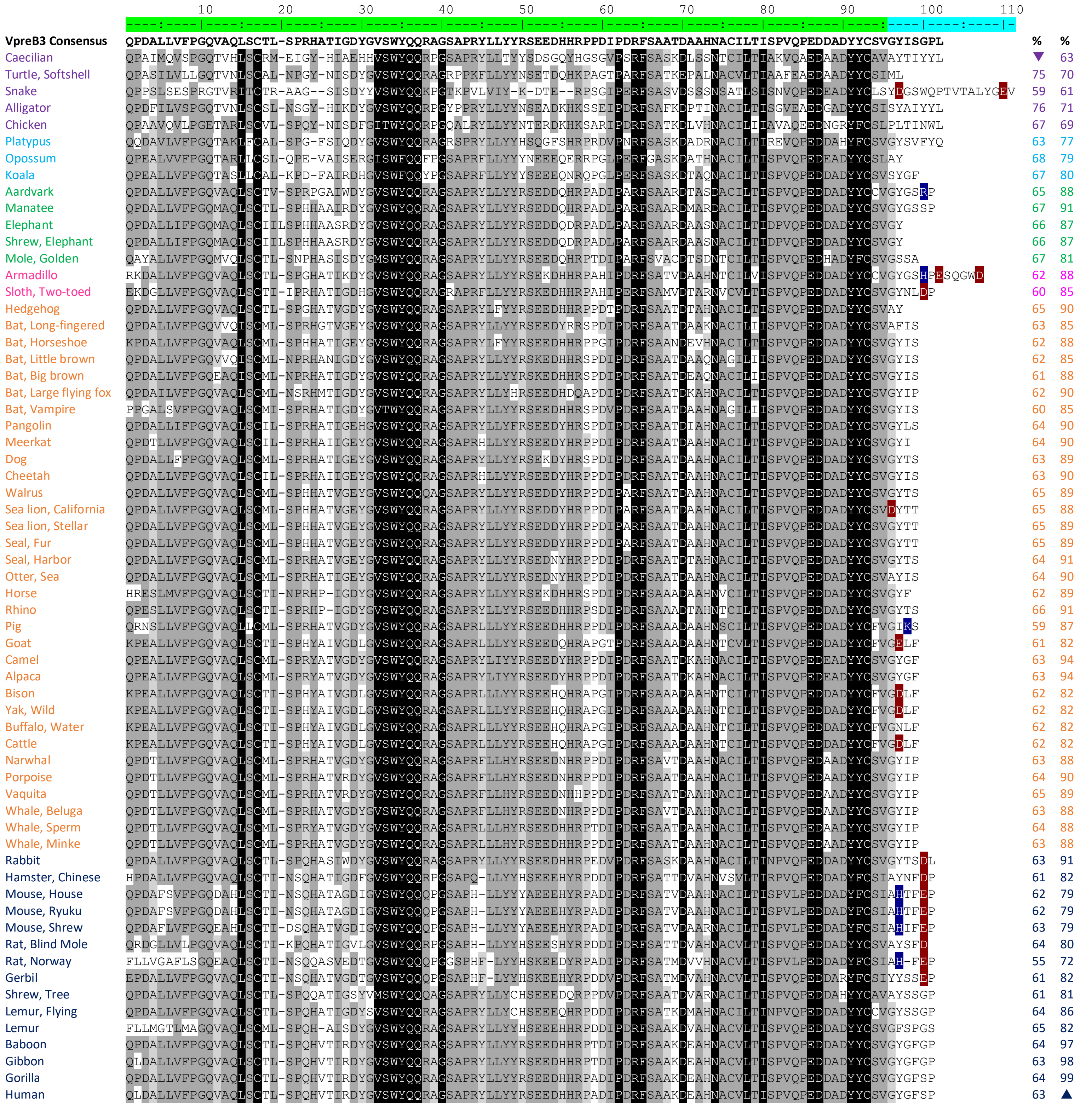

Suppl. Figure 5

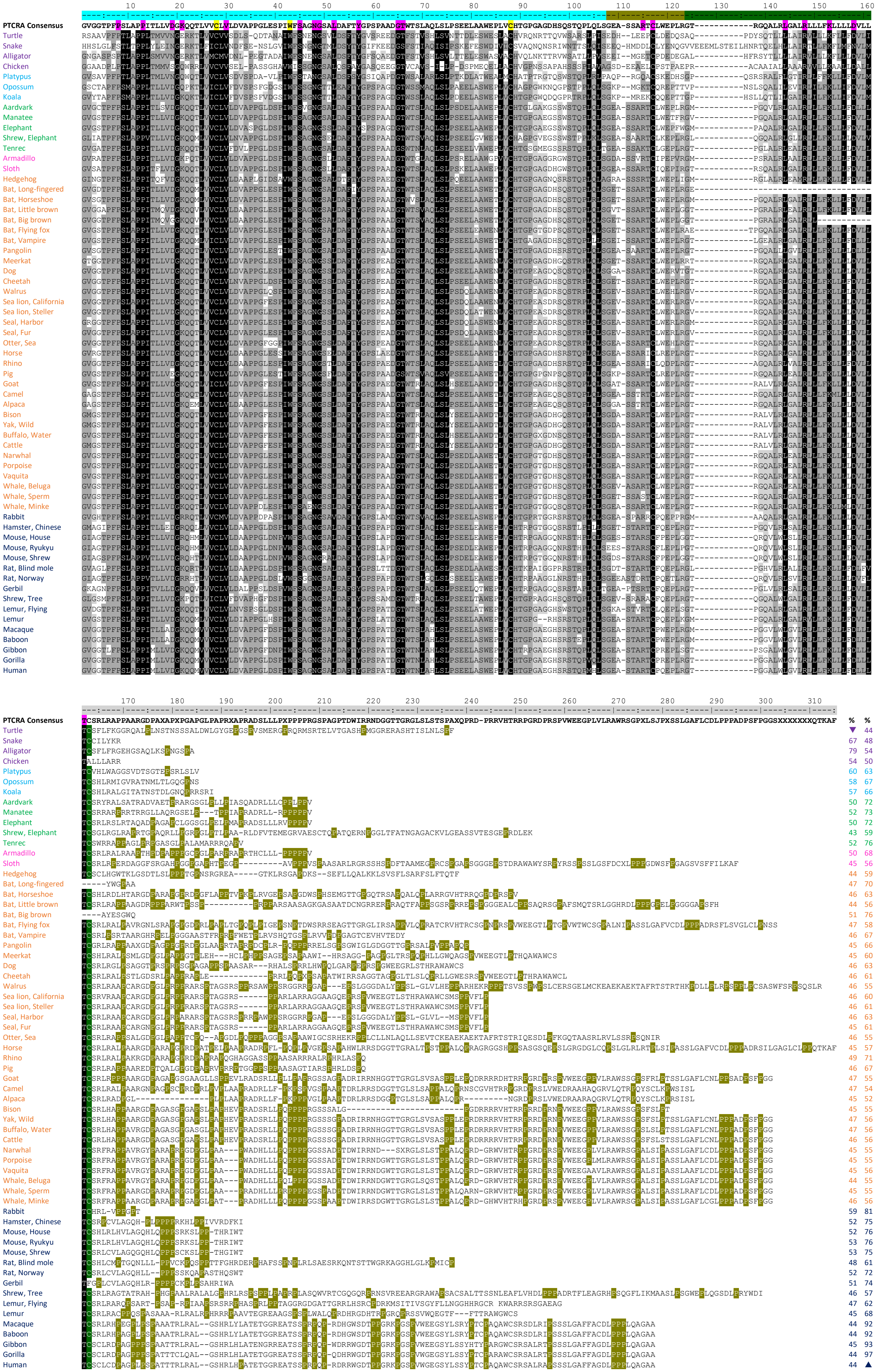

Suppl. Figure 6

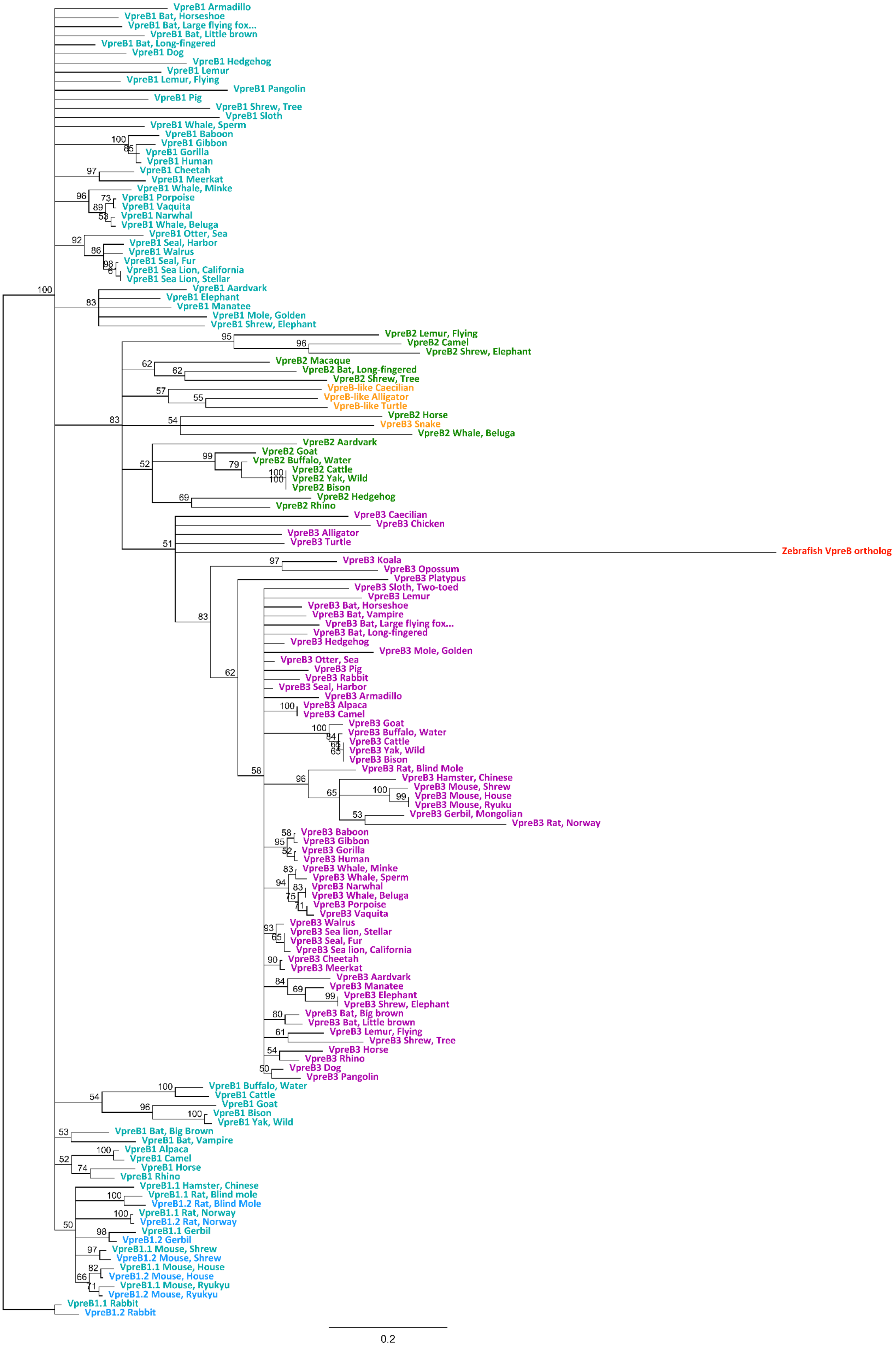

Suppl. Figure 7

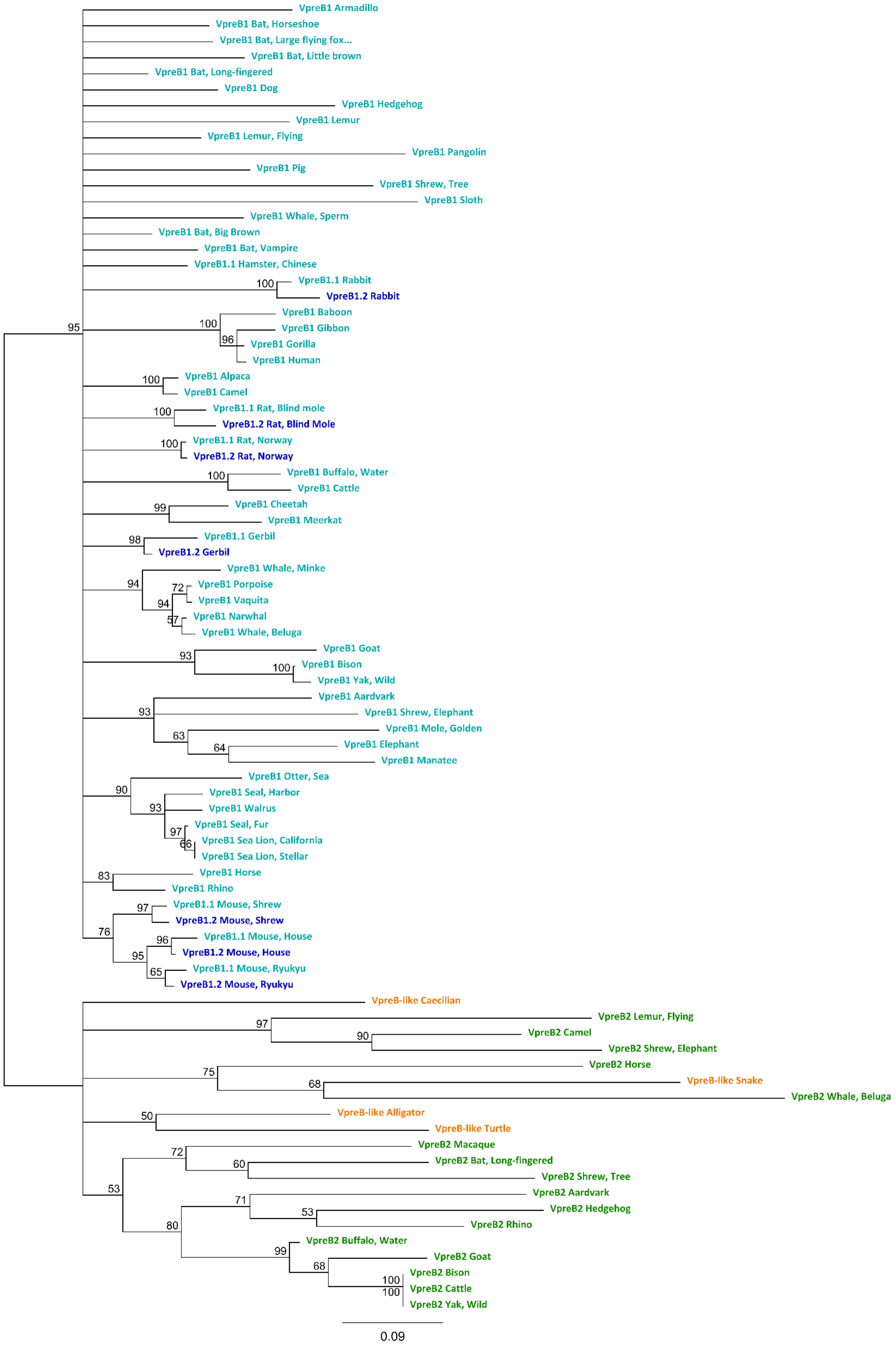

Suppl. Figure 8

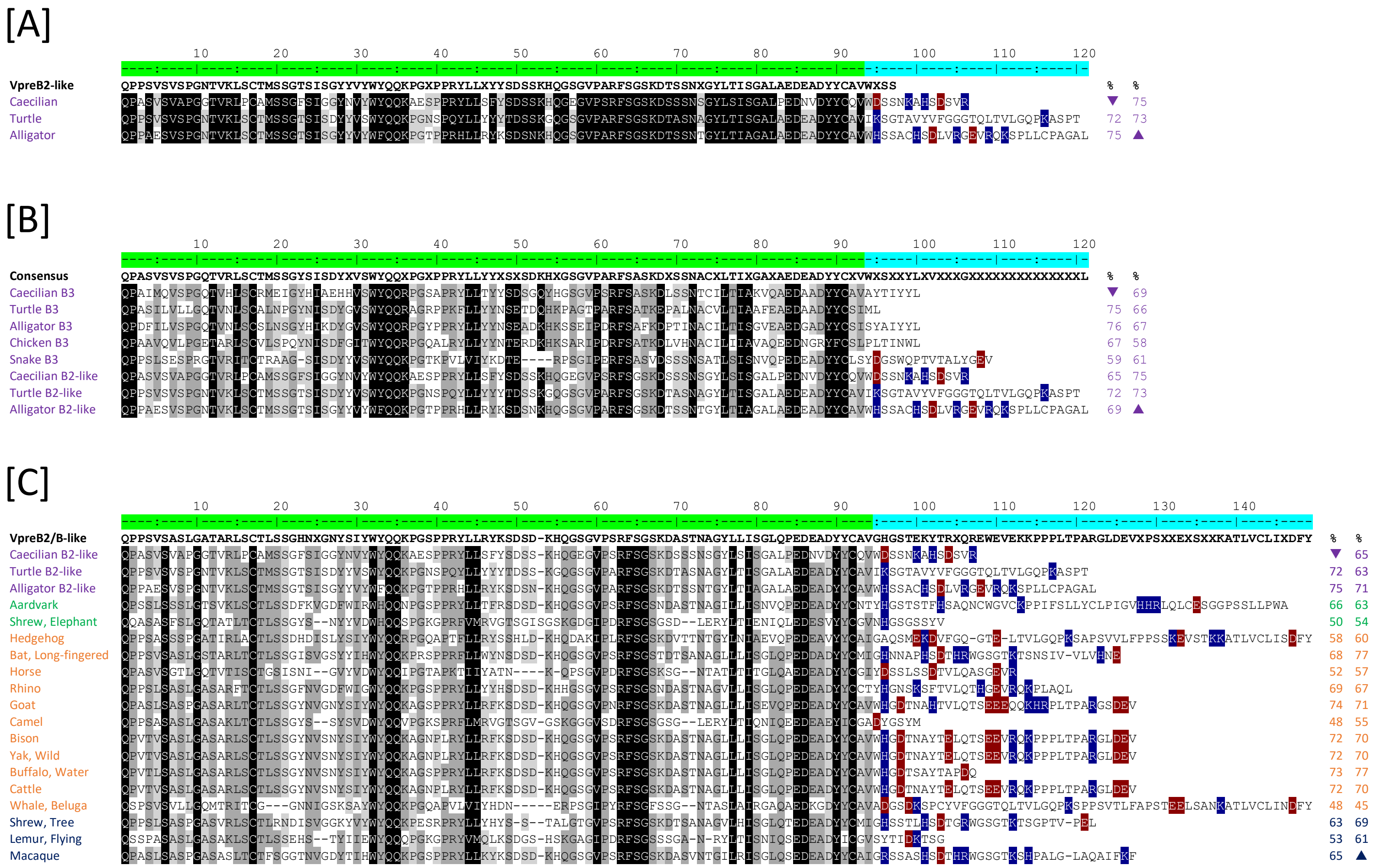

Suppl. Figure 9

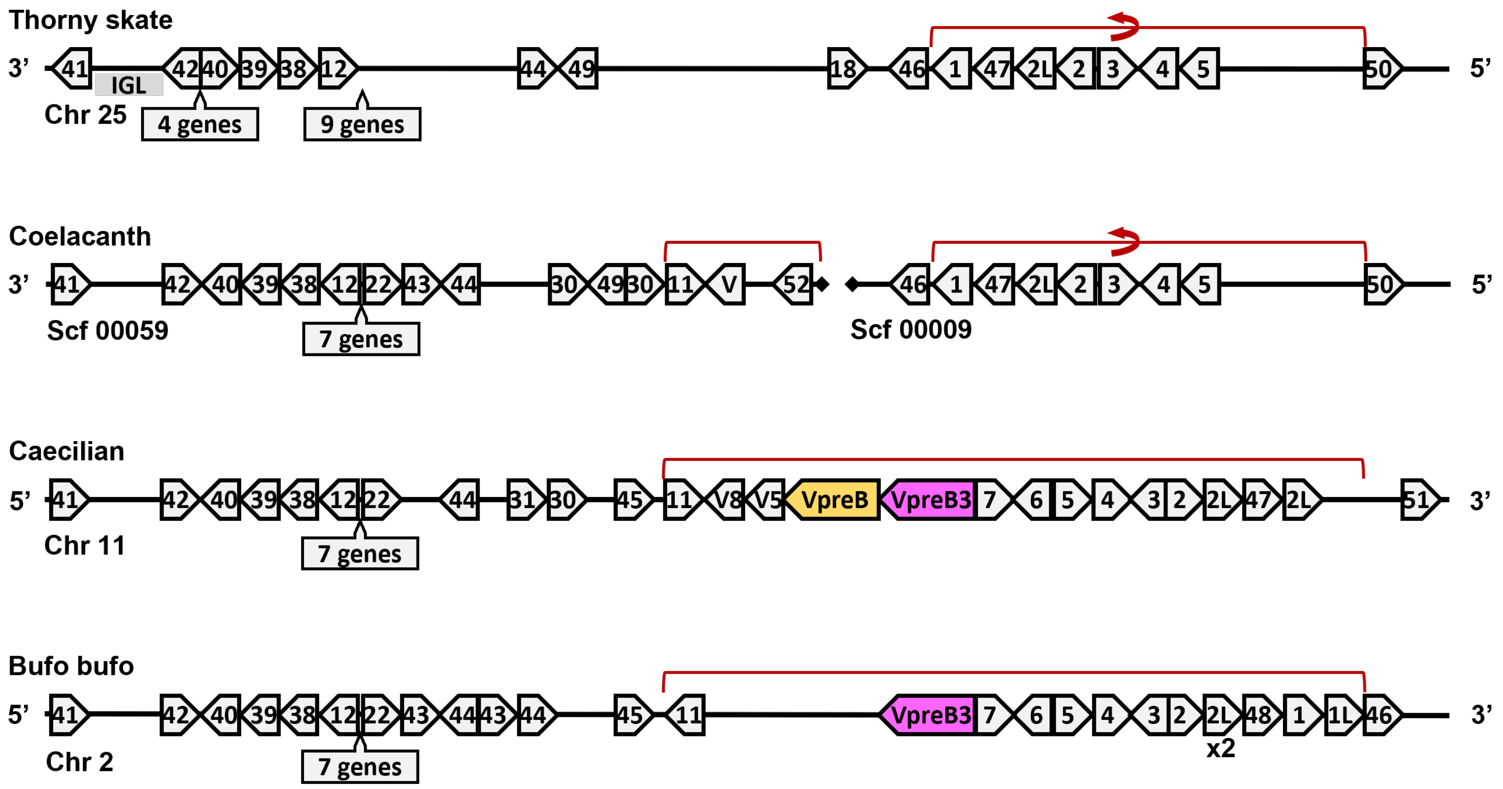

Suppl. Figure 10

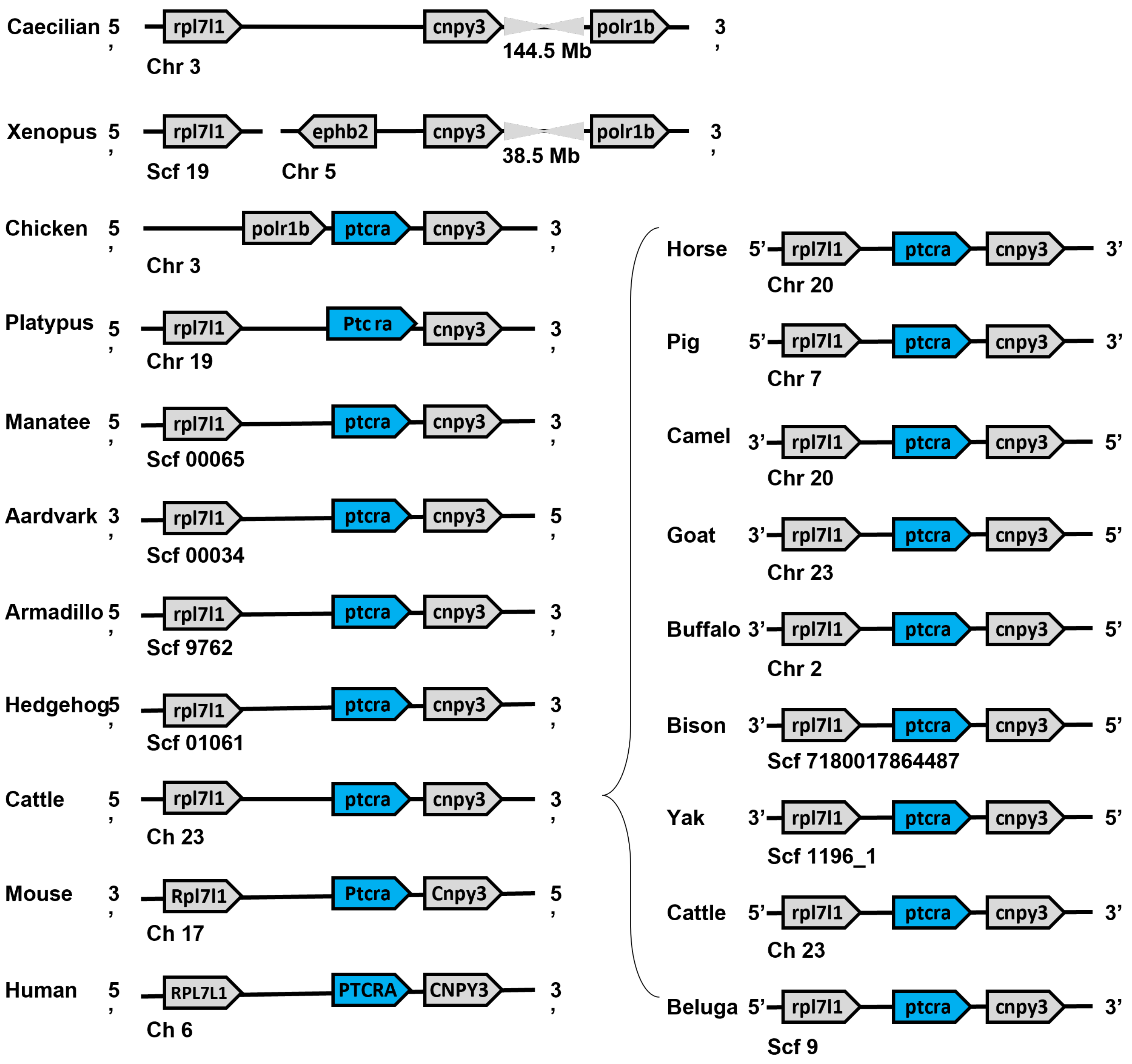

Supplement: Supplementary file 5 [file DataSheet_1.pdf]
